# Automatic Prediction of Molecular Properties Using Substructure Vector Embeddings within a Feature Selection Workflow

**DOI:** 10.1021/acs.jcim.4c01862

**Published:** 2024-12-23

**Authors:** Son Gyo Jung, Guwon Jung, Jacqueline M. Cole

**Affiliations:** † Cavendish Laboratory, Department of Physics, University of Cambridge, J. J. Thomson Avenue, Cambridge CB3 0HE, U.K.; ‡ ISIS Neutron and Muon Source, STFC Rutherford Appleton Laboratory, Harwell Science and Innovation Campus, Didcot, Oxfordshire OX11 0QX, U.K.; § Rutherford Appleton Laboratory, Research Complex at Harwell, Harwell Science and Innovation Campus, Didcot, Oxfordshire OX11 0FA, U.K.; ∥ Scientific Computing Department, STFC Rutherford Appleton Laboratory, Harwell Science and Innovation Campus, Didcot, Oxfordshire OX11 0QX, U.K.

## Abstract

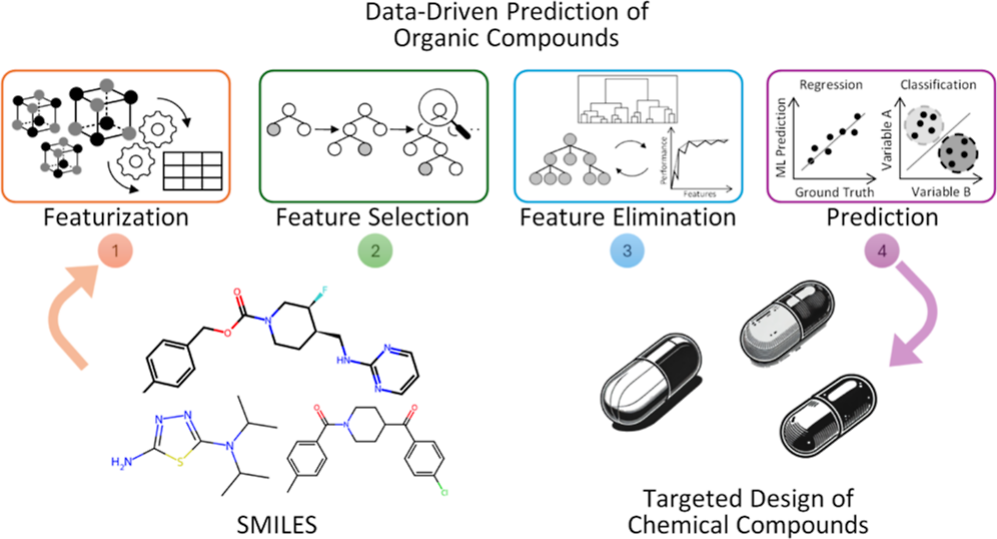

Machine learning (ML) methods provide a pathway to accurately predict molecular properties, leveraging patterns derived from structure–property relationships within materials databases. This approach holds significant importance in drug discovery and materials design, where the rapid, efficient screening of molecules can accelerate the development of new pharmaceuticals and chemical materials for highly specialized target application. Unsupervised and self-supervised learning methods applied to graph-based or geometric models have garnered considerable traction. More recently, transformer-based language models have emerged as powerful tools. Nevertheless, their application entails considerable computational resources, owing to the need for an extensive pretraining process on a vast corpus of unlabeled chemical data sets. To this end, we present a semisupervised strategy that harnesses substructure vector embeddings in conjunction with a ML-based feature selection workflow to predict various molecular and drug properties. We evaluate the efficacy of our modeling methodology across a diverse range of data sets, encompassing both regression and classification tasks. Our findings demonstrate superior performance compared to most existing state-of-the-art algorithms, while offering advantages in terms of balancing model accuracy with computational requirements. Moreover, our approach provides deeper insights into feature interactions that are essential for model interpretability. A case study is conducted to predict the lipophilicity of chemical molecules, exemplifying the robustness of our strategy. The result underscores the importance of meticulous feature analysis and selection over a mere reliance on predictive modeling with a high degree of algorithmic complexity.

## 1 Introduction

Data-driven methodologies have reached to the forefront of materials discovery owing to the advent of big-data initiatives and the expanding accessibility of chemical data sets. Notably, machine-learning (ML) techniques have garnered widespread attention in materials science for their ability to discern intricate chemical structure–property relationships, far exceeding the capabilities of traditional manual analyses. Their computational efficiency in predicting molecular properties affords ML substantial advantages in materials engineering and drug discovery endeavors. ML models have showcased their proficiency in accurately predicting chemical structures and properties,^1−3^ in addition to enabling the automatic characterization of chemical materials across a range of spectra.^4^ The application of a multifidelity modeling strategy, which integrates both density functional theory (DFT) calculations and experimental measurements, exemplifies another sophisticated modeling approach to materials-property prediction across different research domains.^5,6^ Such advancements exemplify the transformative impact of ML to enhance the material-screening process, enabling the exploration of vast chemical landscapes and the identification of novel materials within highly complex feature spaces with an efficiency that is unattainable through conventional design-to-device processes.

In computational materials science, ML approaches use materials informatics, which often include electronic-structure calculations.^2,7^ The generic workflow begins by transforming chemical information into a machine-readable format via feature descriptors. These descriptors encapsulate essential chemical attributes and are used for training predictive models. The utility of these models spans two main applications: (i) predicting the properties of unseen chemical materials in a regression task, and (ii) determining the class or category of materials within a classification context. Additionally, recent ML models have emphasized the automatic extraction of features using graph neural network (GNN)-based approaches, which encode the chemical connectivity of molecular structures.^8−11^ Such techniques provide a universal representation of chemical materials, albeit supervised training of GNNs for molecular property prediction is often constrained by the limited availability of labeled data. Consequently, there has been a surge in the popularity of unsupervised or self-supervised learning frameworks that utilize large unlabeled data sets.^12^

In the domain of organic materials, ML models use the SMILES (Simplified Molecular Input Line-Entry System) representation of chemical molecules.^13^ SMILES serves as a chemical notation system that enables a precise specification of molecular structures through natural grammar. The adoption of learning techniques on SMILES has been widespread for predicting molecular properties, owing to the succinct representation that it provides. Notably, SMILES explicitly encapsulates the presence of chemical elements, diverse bond types, branches, aromaticity, cyclic structures, and local chirality. Such a representation has paved the way for molecular property prediction through language models within the self-supervised setting.^14^

Recently, significant progress has been made in various downstream natural-language-processing tasks by transformer-based language models that are pretrained on extensive unlabeled data sets. To this extent, Ross et al.^15^ introduced molecular embeddings through the training of a MOlecular Language transFORMER (MOLFORMER) that features a linear attention mechanism. This model was trained on sequences of SMILES strings that correspond to a corpus of 1.1 billion unlabeled molecules. Their study illustrated the efficacy of molecular-representation learning and achieved competitive performance against other supervised and unsupervised language models and GNNs. These findings offer promising indications that large-scale molecular language models can capture sufficient chemical and structural information to predict a range of molecular properties through their fine-tuning on a data set that is specific to each task. It is noteworthy that studies have not found any discernible shortcomings in SMILES compared to alternative chemical notation systems, such as self-referencing embedded strings,^16^ in terms of optimization capability and sample efficiency in molecular optimization tasks within the learned representation space.^17^

However, there are certain issues associated with large-scale molecular language models or other modeling methodologies that rely on a vast corpus of unlabeled data within the unsupervised or self-supervised framework. The main drawback is the demand for extensive computational resources. For instance, MOLFORMER^15^ is regarded as an efficient model that is scalable due to its use of adaptive batch bucketing, parallelization, and a linear attention mechanism. Its pretraining process used 16 graphics processing units (GPUs) and required 208 h to complete 4 epochs of loss reduction with a batch size of 1600 molecules per GPU. Without bucketing and linear attention, batch sizes would be constrained to fewer than 50 molecules per GPU, necessitating over 1000 GPUs.

Here, we implement a gradient-boosted and statistical feature-selection (GBFS) workflow that has been customized for materials-property predictions.^18^ Our GBFS workflow integrates a distributed gradient-boosting framework, together with exploratory data and statistical analyses and various strategies to mitigate multicollinearity within a data set. These techniques collectively identify a subset of features that are highly germane to the target variable or class within a complex feature space, ensuring minimal redundancy and maximal relevance to the target variables or classes. The efficiency of this workflow for predicting materials properties has been demonstrated, especially in the area of inorganic chemistry, through comparisons with DFT calculations and experimental data.^19−22^

The primary objective of this study is to validate the broader applicability of our proposed workflow as a general-purpose tool across various research domains, with a particular emphasis on its utility in organic chemistry and drug discovery. This study illustrates the adaptability of our workflow across different areas of organic chemistry, a capability that has not been previously demonstrated. With enhancements that accommodate organic compounds, our workflow can now be applied more extensively across a range of scientific fields and can be seamlessly integrated with other computational methods, such as vector embeddings. To illustrate the versatility of our methodology, we explored an array of more than 20 diverse material properties, demonstrating that it extends beyond the constraints of specific domains. We enriched our data set by incorporating a variety of sources from the scientific literature, which enables direct comparisons and emphasizes the efficacy of our modeling approach in tackling complex predictive tasks, including those related to quantum mechanical properties. Moreover, we utilized data sets originating from experimental sources. Our models adeptly predict properties using only the SMILES notation as input. This capability is exemplified in a case study on lipophilicity, which showcase the adaptability of our workflow in developing and implementing domain-specific models.

Additionally, we seek to understand the impact of improving the predictability of ML models by incorporating systematic analyses at the feature level. This approach contrasts popular practices that prioritize intricate model development, often at the cost of increased computational demand. Our methodology emphasizes the significance of meticulous feature analysis and selection, as opposed to relying solely on complex modeling techniques. We illustrate that a simple tree-based model trained on SMILES-based features, which have been selected and engineered by our GBFS workflow using a single central processing unit (CPU) such as the AMD Ryzen 9 or Intel Core i9, can produce results that are comparable or superior to those reported in the literature, many of which require multiple CPUs, GPUs or high-performance supercomputers. ML methods or workflows that realize such performance gains while requiring considerably fewer computational resources are vital in light of the global need to achieve energy sustainability. Moreover, our workflow offers valuable insights into feature interactions and their importance in predicting target variables, thus advancing our understanding of material properties.

We further explore methods to improve the predictability of our models by integrating substructure vector embeddings that are generated by an unsupervised ML approach. We adopt the implementation of Jaeger et al.^23^ to learn vector representations of molecular substructures. These vector representations are designed to align in similar directions for chemically related substructures, with the final embeddings being computed as a vector addition of the individual substructures. The underlying concept is that vectors of closely related SMILES representations are positioned close to each other in the vector space. We will demonstrate an enhancement in the predictive performance of our workflow via the inclusion of such substructure vector embeddings. This leads to results that outperform many supervised or unsupervised state-of-the-art benchmarks in molecular-property predictions.

We have provided the code that supports the creation of models applicable to any data set, facilitating the prediction of novel chemical properties. This capability is exemplified through specific implementations for inorganic compounds, as detailed in the work of Jung et al.^18^ In this study, we have enhanced the utility to encompass organic compounds and integrated the use of chemical descriptors derived from an unsupervised learning framework, specifically Mol2Vec.^23^ Consequently, our code surpasses domain-specific constraints and is adaptable for use across various research environments. Furthermore, the integration of Mol2Vec underscores the compatibility of our workflow with established methodologies, demonstrating that our tool is not merely an isolated tool but is designed such that it can be seamlessly integrated into broader research ecosystems.

## 2 Methods

### 2.1 Data Sets

The data sets employed in this study were compiled from diverse literature sources, as summarized in Table 1. Among the data sets used is the quantum-chemistry QM9 data set,^24^ which is a subset of the GDB-17 database^25^ and comprises about 130,000 stable organic chemical compounds. These compounds consist of H, C, F, N, and O atoms, with a maximum of 9 heavy atoms in each molecule. The QM9 data set also contains 12 molecular properties that have been computed for each molecule at the B3LYP/6-31G(2df,p) level of quantum chemistry. These properties are summarized in Table 2. In contrast to studies by others, we did not include geometric properties such as atomic coordinates in our features. Instead, we limited our features to only those that can be derived from the SMILES representation. This decision was made considering that comprehensive three-dimensional (3D) molecular geometric information is often absent in the literature; so, removing any reliance on such information streamlines the materials-property prediction process. Such a methodological choice also allows us to evaluate the efficacy of our modeling approach, given that predicting quantum-chemical properties solely from SMILES representation is considered nontrivial.

**Table 1:** Summary of Datasets Used in This Study

subject category	name of data set	no. & type of tasks		no. of compounds	description of data set contents
quantum mechanics	QM9	12	regression	133,885	12 quantum mechanical calculations of organic molecules
physical chemistry	ESOL	1	regression	1127	water solubility
	FreeSolv	1	regression	641	hydration free energy of molecules in water
	lipophilicity	1	regression	4200	octanol–water distribution coefficient of molecules
photovoltaics	PE	1	regression	29,978	photovoltaic efficiency of organic molecules
biophysics	HIV	1	classification	41,126	ability of drugs to inhibit HIV replication
	BACE	1	classification	1512	binding results for a set of inhibitors for β-secretase 1
physiology	BBBP	1	classification	2038	blood–brain barrier penetration ability of compounds
	Tox21	12	classification	7830	toxicity measurements of compounds on 12 different targets
	SIDER	27	classification	1426	side effect of drugs on 27 different organ classes
	ClinTox	2	classification	1476	clinical trial toxicity and FDA approval status of drugs

**Table 2:** Chemical Properties Contained within the QM9 Dataset

no	property	description
1	μ	dipole moment
2	α	isotropic polarizability
3	*E*_HOMO_	energy of highest occupied molecular orbital (HOMO)
4	*E*_LUMO_	energy of least unoccupied molecular orbital (LUMO)
5	*E*_gap_	gap (*E*_LUMO_ – *E*_HOMO_)
6	⟨*R*^2^⟩	electronic spatial extent
7	ZPVE	zero point vibrational energy
8	*U*_0_	internal energy at 0 K
9	*U*	internal energy at 298.15 K
10	*H*	enthalpy at 298.15 K
11	*G*	free energy at 298.15 K
12	*C*_*v*_	heat capacity at 298.15 K

Other data sets employed in our study include: (i) estimated solubility (ESOL),^26^ a data set of water solubility data; (ii) Free Solvation Database (FreeSolv),^27^ a data set of experimentally measured and calculated hydration free energy of small molecules in water; (iii) lipophilicity,^28−30^ a data set of experimental results on the octanol–water distribution coefficient of molecules, which is a feature of a drug molecule that concerns membrane permeability and solubility; (iv) photovoltaic efficiency (PE),^31^ a data set of photovoltaic efficiency of organic molecules; (v) human immunodeficiency virus (HIV),^32^ a data set sourced from the Drug Therapeutics Program AIDS Antiviral Screen to test the ability of drugs to inhibit HIV replication; (vi) β-secretase (BACE),^33^ a data set of experimental binding results for a set of inhibitors for β-secretase 1; (vii) blood–brain-barrier penetration (BBBP),^34^ a data set about the blood–brain barrier penetration or permeability properties of chemical compounds (viii) toxicity-21 (Tox21),^35,36^ a data set of toxicity measurements on 12 different targets, including nuclear receptors and stress response pathways; (ix) Side Effect Resource (SIDER),^30,37^ a data set of marketed drugs and adverse drug reactions, delineating the side effect of drugs on 27 different organ classes; (x) Clinical Toxicity (ClinTox),^30,38−40^ a data set consisting of data on clinical trial toxicity and Food and Drug Administration (FDA) approval status of drugs. Once again, we confine our features to those that can be generated from SMILES representations alone.

The data-splitting method employed for these data sets precisely replicates that of Ross et al.,^15^ who implemented a train-to-test split ratio of ca. 9:1. The training and the test sets are, therefore, identical for each task that we perform unless explicitly stated otherwise. Consequently, this enables a like-for-like comparison to the literature benchmarks, and the performance metrics reported in this study offer a direct comparison to the findings of Ross et al. The sole exception to this procedure is in a case study that examines the lipophilicity of chemical molecules in Section 3.2, where we intentionally reduced the size of the training set to impose more rigorous criteria for model assessment. We chose to expand the size of that test set to include a broader range of chemical molecules, increasing the proportion of the test set to 20% of the data set, up from its original 10%. This adjustment allows for a more thorough assessment and comprehensive evaluation of our proposed approach. Moreover, we have utilized the scaffold splitting method for the analyses involving the following data sets: BBBP, Tox21, ClinTox, HIV, Bace, and SIDER. A scaffold splitting strategy, which is based on molecular substructures, imposes a more stringent test of the model’s ability to generalize to new, unseen data.

More specifically, to evaluate the generalization capabilities of our ML models beyond the training distribution, we employed a scaffold splitting strategy as delineated by Ramsundar et al.^41^ This method involves grouping molecules based on their molecular substructures, or scaffolds, as defined by Bemis and Murcko.^42^ The most prevalent scaffolds are placed into the training set, while the validation and test sets are composed of structurally distinct molecules. This scaffold splitting approach presents a significant challenge to learning algorithms, far exceeding that of random splits, by guaranteeing that structurally unique subsets are explicitly separated. The implementation of such a splitting methodology is available in the RDKit library.^43^

### 2.2 Gradient-Boosted Statistical Feature Selection Workflow

**Figure 1:**
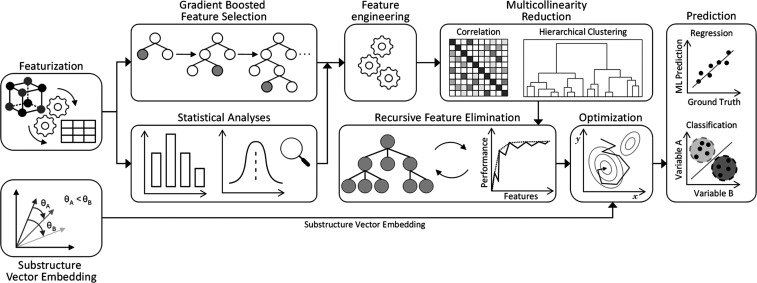
Operational workflow of our GBFS-Mol2Vec pipeline. For an in-depth explanation of the processes involved, the reader is referred to Jung et al.^18^ Adapted with permission from ref (18), Copyright 2023 AIP Publishing.

The GBFS-Mol2Vec implementation that we employed in this work is portrayed in Figure 1. Its feature-generation phase uses an extensive set of descriptors to construct a high-dimensional feature vector, with SMILES strings as inputs. These descriptors encompass various feature representations, such as Morgan fingerprints, RDKit molecular fingerprints, ElemNet, Maccs keys, element property fingerprints, atom pair counts, and extended-connectivity circular fingerprints.^43−52^ By employing all these descriptors, each material employed in this study was assigned a base feature vector (i.e., prior to feature engineering) with a length of ca. 11,000 through the concatenation of outputs from these descriptors. These resulting feature vectors served as inputs to our GBFS workflow. The workflow identifies a subset of features that offer maximum loss reduction and minimal redundancy when training ML models for specific tasks or material-property predictions. Substructure vector embeddings were generated using a molecule-to-vector (Mol2Vec) technique,^23^ which takes SMILES strings as inputs. These embeddings were then directly incorporated into the model optimization phase, being concatenated with the descriptor features that were created via our GBFS workflow.

Our originally designed GBFS workflow then integrates a core sequence of steps that are designed to optimize feature selection and model training for ML applications. This involves a gradient-boosting framework, which identifies a subset of features that are most salient to the target variable or classification task. This is followed by statistical analyses that evaluate the significance of these features in relation to the target variable or class. Subsequently, a feature-engineering process introduces additional features that enhance model input data. To minimize feature redundancy, a two-step multicollinearity reduction involving both correlation and hierarchical clustering analyses is applied. The process also includes recursive feature elimination (RFE), which systematically removes less important features for a chosen performance metric. Lastly, Bayesian optimization is used to perform hyperparameter tuning of the final predictive ML model.

While a detailed description of each component of our original GBFS workflow (i.e., without substructure vector embeddings) has been extensively outlined by Jung et al.,^18^ here we succinctly summarize its key attributes to clarify our methodology. Our modeling approach provides clear advantages by leveraging a systematic framework that minimizes human intervention at both the feature selection and model development stages. Initially, we evaluate the contribution of each feature through an analysis of loss reduction or variance gain, employing first and second order derivatives of the objective function as gradient boosting decision trees (GBDTs) are developed. Concurrently, we perform a series of statistical tests and analyses that are rooted in probability theory and information theory. These dual processes effectively isolate the most pertinent features for a given target variable or target classes. These key features subsequently serve to generate additional features. Typically, a brute force method is employed that requires no domain-specific knowledge; however, manual intervention can be introduced at this stage to refine feature engineering. Following this, the most relevant and statistically significant features, along with those newly engineered, are scrutinized for multicollinearity.

The process of addressing multicollinearity begins with the elimination of highly correlated features, adhering to a pre-established correlation threshold. This is augmented by a hierarchical clustering analysis that groups similar features, setting a linkage threshold that enables the algorithm to automatically select a representative feature from each cluster. This approach is predicated on the principle that a single representative feature can encapsulate the essential information on a cluster, thus efficiently condensing the feature space without sacrificing critical data. Following this, RFE is performed, employing a greedy-based search strategy to methodically prune features until an optimal feature set is determined, based either on achieving the desired feature count or ensuring no degradation in model performance occurs. Concurrently, permutation importance analysis is conducted by randomly altering the values of individual features to assess their impact on performance metrics, further refining the model’s robustness and predictive accuracy.

This rigorous process culminates in a refined subset of features, which is used to conduct Bayesian optimization. The optimization phase autonomously determines the most efficient model architecture using only the training set, thereby eliminating the need for human intervention or oversight throughout this stage. Once optimized, the final predictive model undergoes evaluation using the test set—this constitutes the sole instance where this data set is employed. This comprehensive strategy guarantees that the selected features contribute optimally to the predictive accuracy of our ML models, while effectively mitigating the impacts of high correlations and redundancy among the input features. By significantly simplifying the complexity of the feature space, our approach addresses potential overfitting issues and inherently carries out regularization to promote model generalization. This highlights the sophistication and reliability of our systematic analytical methodology.

It is worth noting that variability in the selection of features (i.e., the feature profile) by our GBFS workflow is influenced by the training data. Such variability is particularly pronounced with smaller data sets, which may not sufficiently capture the chemical diversity, while in larger data sets, this variation tends to be minimal or negligible. This behavior is anticipated and represents a common bottleneck for various ML techniques. Additionally, this variability can be exacerbated by a degree of randomness inherent in the feature reduction stages of the workflow, particularly during multicollinearity reduction, where features that are highly correlated are eliminated randomly. Nevertheless, users have the option to intervene and make more selective choices during this process.

### 2.3 Evaluation Metrics for Analysis

For the regression analysis, we consider the mean absolute error (MAE), the mean squared error (MSE) and the coefficient of determination that is defined as the square of the Pearson correlation coefficient, *R*
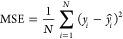
1
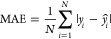
2
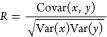
3where *y* and  are the true and predicted values, respectively, over *N* number of samples;  is the covariance between *x* and *y*;  and  are the variance of *x* and *y*;  and  are the mean of *x* and *y*, respectively. The range of *R* is [−1, 1], reflecting the linear tendency of a quantity to change as another quantity varies.

For the classification analysis, we consider the area-under-the-curve of the receiver-operating-characteristic curve (AUC-ROC), the accuracy and the *F*1-score, where the latter is defined as the harmonic mean of the precision and recall, as follows
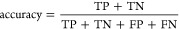
4
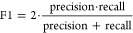
5
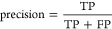
6
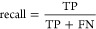
7where TP and TN are true positive and true negative, and FP and FN are false positive and false negative, respectively. For each regression or classification task that we undertake, we maintain consistency in the evaluation of our models by quoting the performance metric specified in the existing literature.

## 3 Results and Discussion

### 3.1 Evaluation of Model Performance

We assessed the efficacy of our modeling technique using 5 data sets across 16 regression tasks, and 6 data sets across 44 classification tasks. Our findings are compared with those of state-of-the-art reports in the following discussion.

#### 3.1.1 Regression Tasks

Table 3 presents the comparative performance of various material-property predictions from different ML models on the QM9 test set, as judged by reports of MAE values. Each row corresponds to a specific material property, while each column represents a different ML model utilized for the predictions. The ML models evaluated comprise: an attentive-FP (A-FP)^53^ model; a graph convolutional (GC) network;^54^ a multitask neural network encoding the Coulomb Matrix (CM);^55^ a deep-tensor neural network (DTNN);^56^ a message-passing network (MPNN);^11^ Chemical RoBERTa (ChemBERTa);^14^ MOLFORMER (QM9 only);^15^ MOLFORMER-XL;^15^ GBFS; GBFS-Mol2Vec. Here, GBFS refers to our original GBFS workflow,^18^ while GBFS-Mol2Vec denotes our GBFS workflow that incorporates substructure vector embedding.

**Table 3:**
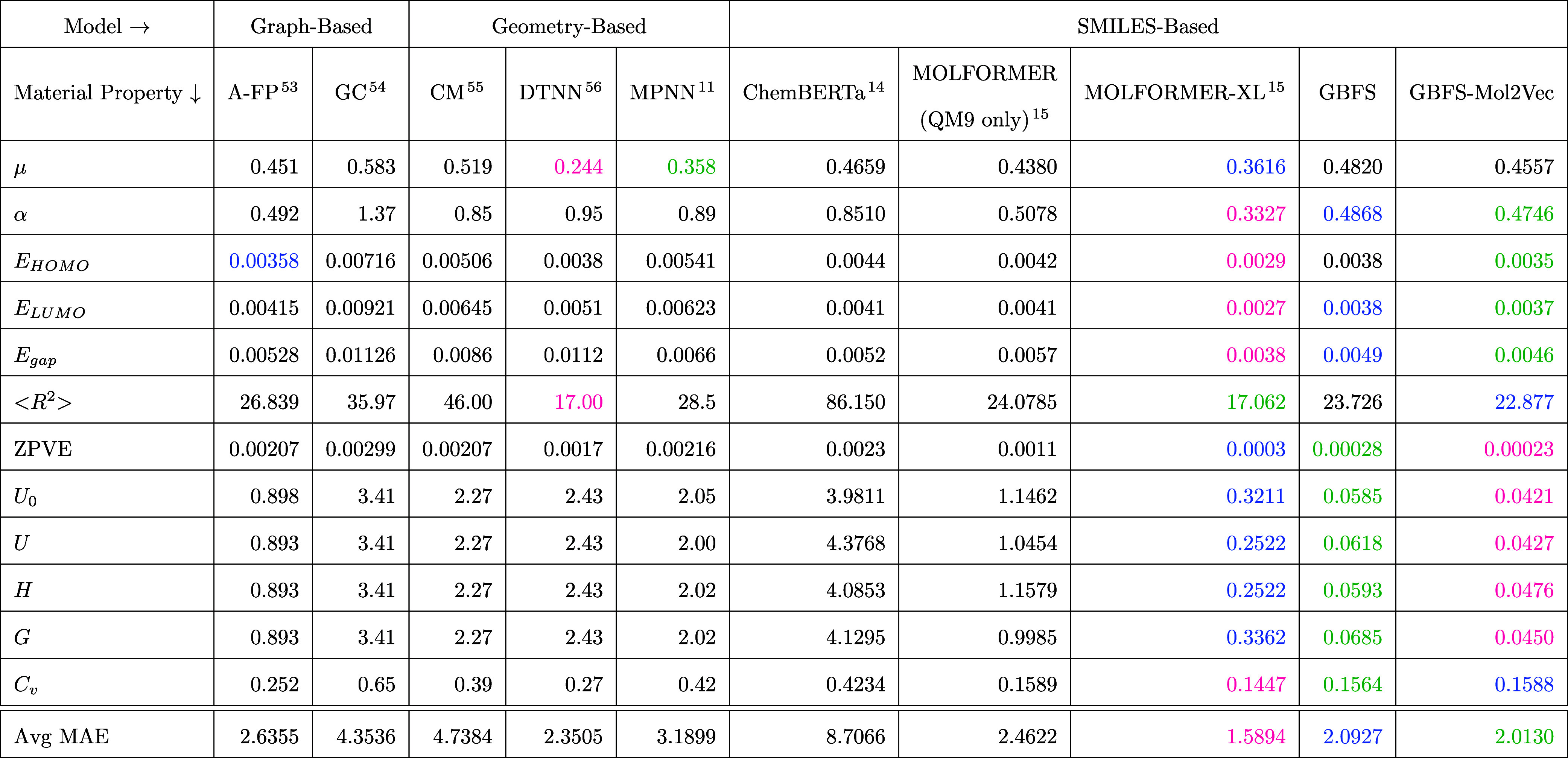
Performance of Various ML Models on the QM9 Test Set^a^

a MAE values are color-coded in shades of pink, green, and blue to denote the top-performing, second-best, and third-best models, respectively. Performance benchmarks were obtained from multiple sources.^15,30,53,73^

In general, MOLFORMER-XL, GBFS-Mol2Vec, and GBFS exhibit the most favorable average MAE values across the spectrum of material properties, which indicates their superior predictive capabilities compared to the other evaluated models. Conversely, models such as CM and ChemBERTa display higher average MAE values across the 12 properties, suggesting relatively inferior performance in predicting the quantum-chemical properties of organic molecules. Notably, our proposed modeling approach outperforms the MOLFORMER (QM9 only) model in all prediction tasks. The MOLFORMER (QM9 only) model is a molecular-language transformer model trained solely on the QM9 data set, while MOLFORMER-XL has been pretrained on a data set comprising ca. 1.1 billion molecules; these include data from PubChem^57^ and ZINC.^58^ It is unsurprising to observe that MOLFORMER-XL achieves the lowest average MAE, although it is noteworthy that the GBFS-based models appear to perform with greater accuracy than the MOLFORMER approach when the data set is limited to QM9 alone. There exists a clear trade-off between model predictability and the volume of data employed. It is important to remember that the use of more data results in higher computational demands. This is not to disregard the advantages of employing self-supervised learning with a vast corpus of unlabeled data. However, it is imperative to select the appropriate modeling method according to energy-sustainability considerations, as well as the practical availability and use of computational resources for these prediction tasks.

A closer examination of these MAE statistics reveals that the DTNN and MPNN models afford commendable performance in predicting the dipole moment (μ), with respective MAE values of 0.244 and 0.358 D. Similarly, the DTNN model exhibits the lowest MAE in predicting the electronic spatial extent (⟨*R*^2^⟩). These results are anticipated given that both models are geometry-based, integrating 3D molecular geometric data into their feature sets. Furthermore, we observe that our GBFS-based models both achieve markedly lower MAE values than alternative methods when predicting internal energies at 0 K and 298.15 K (*U* and *U*_0_), enthalpy at 298.15 K (*H*), and free energy at 298.15 K (*G*). These MAE values are also on par with those obtained by the higher-order GNN model developed by Morris et al.^59^ referred to as 1-2-3-GNN, who achieved MAE values of a similar magnitude. Nevertheless, it is worth noting that the more expressive and potent 1-2-3-GNN-based models are difficult to scale,^60^ even when compared to MOLFORMER-XL, which employs a linear attention mechanism to ensure linear time complexity.^15^ Despite the predictive capabilities of the 1-2-3-GNN, we have decided to exclude this model from our comparative analysis hereafter, as it is well-documented that such networks are challenging to scale.

Table 3 also offers valuable insights into the relative efficacy of diverse models in forecasting various material properties. Forecasting ability is crucial when choosing the most appropriate model for specific applications in materials science. It is essential to highlight that the selection of an ML model can profoundly influence the precision of material-property predictions. Overall, our GBFS and GBFS-Mol2Vec models emerge as promising modeling methodologies in the prediction of quantum-mechanical calculations of organic molecules; cf. Statistics in Table 3 demonstrate their competitive performance across numerous material properties and models, and do so despite the fact that they do not require significant computational resources.

Before delving further into our analysis, it is necessary to be aware of conformational effects in the context of molecular chemistry.^61^ These effects manifest as variations in atomic arrangements within a molecule, stemming from different spatial orientations. While spectroscopic data can offer insights into these conformational effects by representing a Boltzmann average of all conformers present in a solution, SMILES strings lack the capacity to account for such variations. Given that this study does not differentiate between cases involving single or multiple conformations in the calculations, nor does it address potential modifications to the conformational ensemble due to interactions with the environment, it is imperative to consider these factors when citing or referencing the methodology and the findings presented herein. This is pertinent not only to this study but also to other research endeavors that employ SMILES representations; subject to the caveat that this may not be applicable to certain properties such as solubility, which is a property of a molecule rather than its specific conformers.

In addressing the limitations of SMILES representations for capturing the diverse spatial orientations and conformations of molecules, it becomes critical to evaluate the implications for predictive accuracy in molecular modeling. The inherent inability of SMILES to account for conformational variability restricts the capability of ML models to discern spatial variations. This limitation is particularly detrimental for predicting molecular properties that are profoundly influenced by molecular geometry and conformation, such as reactivity, which may exhibit substantial variation with changes in molecular conformation. As SMILES representations lack the capacity to convey information on molecular dynamics or energetically favorable structures, models that rely exclusively on SMILES representations are prone to oversimplifying molecular complexity, potentially leading to inaccurate predictions by generating uniform outputs irrespective of the conformational state of a molecule. This shortfall is notably critical in the fields of pharmaceutical research and materials science, where a comprehensive understanding of a molecule’s conformational ensemble is essential for accurately predicting interactions with biological targets or other chemical entities. Without the ability to account for the various conformations that a molecule may adopt, SMILES-based models are likely to overlook crucial interactions, thus hindering effective drug design and materials synthesis.

Additionally, the lack of environmental consideration further constrains the generalizability of these models. Molecular conformations are susceptible to alterations during interactions with solvents or in complex biological formations. Consequently, without adaptations or additional data to accommodate these interactions, SMILES-based models will tend to underperform when extrapolated beyond the specific conditions or data sets on which they were originally trained. While SMILES representations may suffice for modeling certain properties such as solubility, which is generally less sensitive to precise atomic configurations as mentioned earlier, they are inadequate for applications that demand detailed 3D structural data. Therefore, the utility of SMILES representations is restricted to a subset of molecular properties and should be selectively applied based on the property under examination.

In light of these considerations, it is imperative for researchers to exercise caution in using SMILES representations as the sole structural representation in molecular property modeling. There is a pressing need to incorporate additional data forms that can capture 3D structural details and dynamic conformational changes. Employing techniques that integrate molecular descriptors reflecting 3D spatial or geometric information could substantially enhance the predictive accuracy of ML models. For achieving comprehensive and reliable predictions, a hybrid approach that amalgamates multiple molecular representations is often essential. This topic is suggested for further exploration as a potential avenue for subsequent research. In this study, we exclusively utilize the SMILES representations to facilitate a direct like-for-like comparison with state-of-the-art benchmarks.

Additional analyses were conducted to demonstrate the versatility of the proposed methodology as a general-purpose tool. Table 4 presents an overview of the model performance measured by root-mean-squared error (RMSE) across various regression tasks, encompassing data from repositories about water solubility (ESOL), hydration free energy in water (FreeSolv), and the octanol–water distribution coefficient of molecules (lipophilicity). The models assessed in this study include a range of approaches: GC;^54^ A-FP;^53^ MPNN;^11^ a variant of MPNN known as DimeNet, which integrates spatial interactions within molecules and incorporates directional message passing;^62^ a self-supervised molecular-graph-representation model pretrained using 2D graph topology and 3D conformal geometry (GeomGCL);^63^ a geometry-enhanced molecular representation learning (GEM),^64^ which incorporates atom-bond-angle relations in the graph; MOLFORMER-XL;^15^ and our GBFS and GBFS-Mol2Vec models.

**Table 4:**
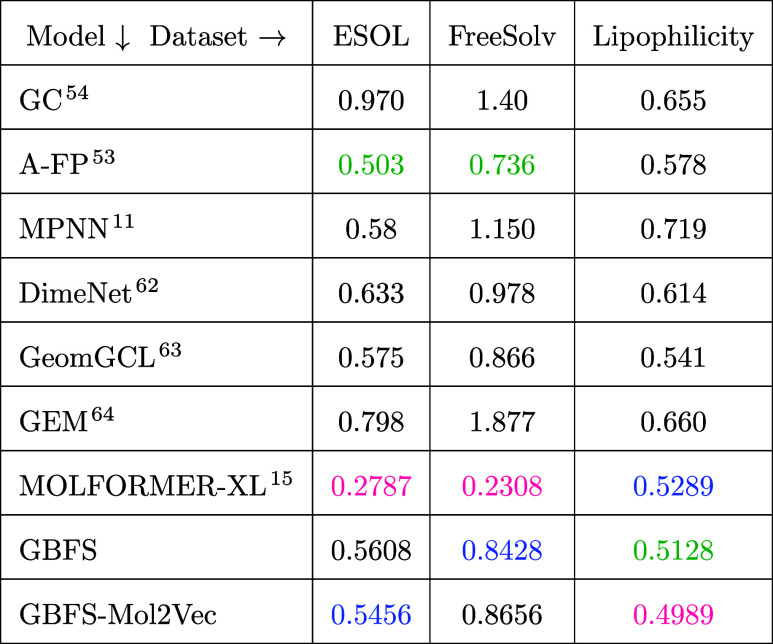
Model Performance on Addition Regression Tasks^a^

a Model performance was assessed using RMSE, with color-coding indicating the top-performing model in pink, second-best in green, and third-best in blue. Performance benchmarks were obtained from various studies.^15,30,53^

For the regression tasks that aim to predict water solubility (ESOL) of molecules or their hydration free energy in water (FreeSolv), the MOLFORMER-XL model displayed the lowest RMSE, followed by the A-FP model and our GBFS or GBFS-Mol2Vec model. The remarkable performance of the MOLFORMER-XL model in these tasks is possibly due to its use of a vast data set that was sourced from PubChem^57^ and ZINC,^58^ as mentioned earlier. Our GBFS-based models performed competitively, positioning between the supervised A-FP and the geometry-informed, self-supervised GeomGCL models. However, geometry-informed models may not be universally applicable or generalizable in scenarios where 3D molecular geometric data are not readily available. This limitation combined with the higher computational requirements of these models could pose challenges, especially for large molecules.

Concerning the regression-based task of predicting lipophilicity, our GBFS-Mol2Vec model achieved the lowest RMSE value, closely followed by GBFS and MOLFORMER-XL. The GBFS-based models and the MOLFORMER-XL model rely exclusively on SMILES representations, and they outperform all other aforementioned models that rely on geometry-informed approaches in the forecast of the octanol–water distribution coefficient of molecules. Furthermore, these results clearly demonstrate the generalizability of our GBFS-based models.

The MSE values corresponding to different ML models employed for the prediction of photovoltaic efficiency (PE) were also analyzed. Table 5 displays the findings which compare six ML models: neural fingerprints (NFPs),^8^ designed to extract data-driven features by employing a back-propagation algorithm; molecular graph convolutional networks (GCNs),^65^ which leverage a deep-learning architecture to represent small molecules as undirected graphs of atoms; both the bidirectional long short-term memory (BiLSTM) and self-attention mechanism BiLSTM (SA-BiLSTM);^66^ and our two GBFS-based models. Among the models evaluated, the GBFS model stands out with the lowest MSE, closely followed by GBFS-Mol2Vec and GCN models. These results suggest that both of our GBFS-based models exhibit excellent performance in predicting photovoltaic efficiency. Conversely, models such as NFP and BiLSTM show relatively higher MSE values, indicating comparatively poorer predictive accuracy for this particular material property. Overall, these findings provide valuable insights into the efficacy of different modeling approaches in the context of photovoltaic efficiency prediction, which requires an analysis of a complex feature space. This may help to explain our analytical findings given that a major challenge in neural-network-based models is the creation of unintuitive features that are often hard to interpret. In contrast, our GBFS-based models do not depend on this type of feature learning process.

**Table 5:**
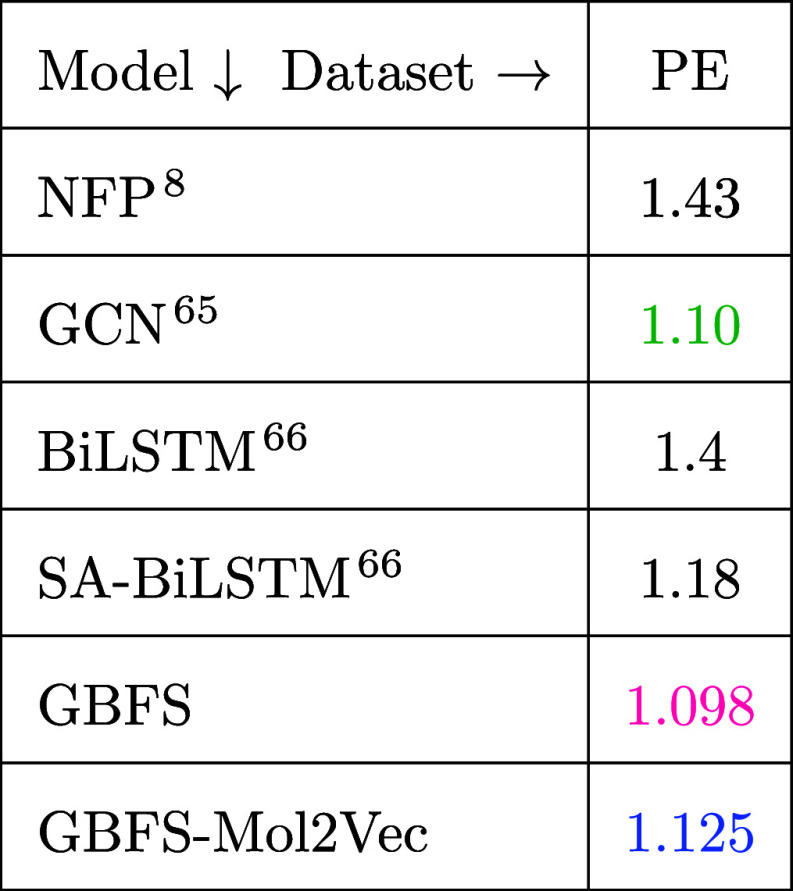
Model Performance on Photovoltaic Efficiency of Organic Molecules^a^

a Model performance was assessed using MSE, with color-coding indicating the top-performing model in pink, second-best in green, and third-best in blue.

Considering the overaching analysis of these regresion tasks, the MOLFORMER-XL model consistently exhibits competitive performance, particularly excelling in predicting water solubility and hydration free energy in water. The GBFS-Mol2Vec model also shows promising results, especially in predicting lipophilicity and photovoltaic efficiency. These findings underscore the effectiveness of self-supervised models, such as the MOLFORMER-XL model, as well as supervised and semisupervised approaches, such as our GBFS and GBFS-Mol2Vec models, in capturing complex molecular properties for diverse research applications in materials science. Such findings demonstrate the versatility and applicability of our proposed methodology.

#### 3.1.2 Classification Tasks

Table 6 presents the AUC-ROC values for various ML models across different classification tasks using scaffold splitting. These tasks employed data sets that inform blood–brain barrier penetration (BBBP), toxicity in the 21st Century (Tox21) on 12 different targets, clinical trial toxicity and FDA approval status of drugs (ClinTox), ability of a drug to inhibit HIV replication (HIV), binding results for inhibitors for β-secretase 1 (BACE), and the side effect of marketed drugs and adverse drug reactions (SIDER).

**Table 6:**
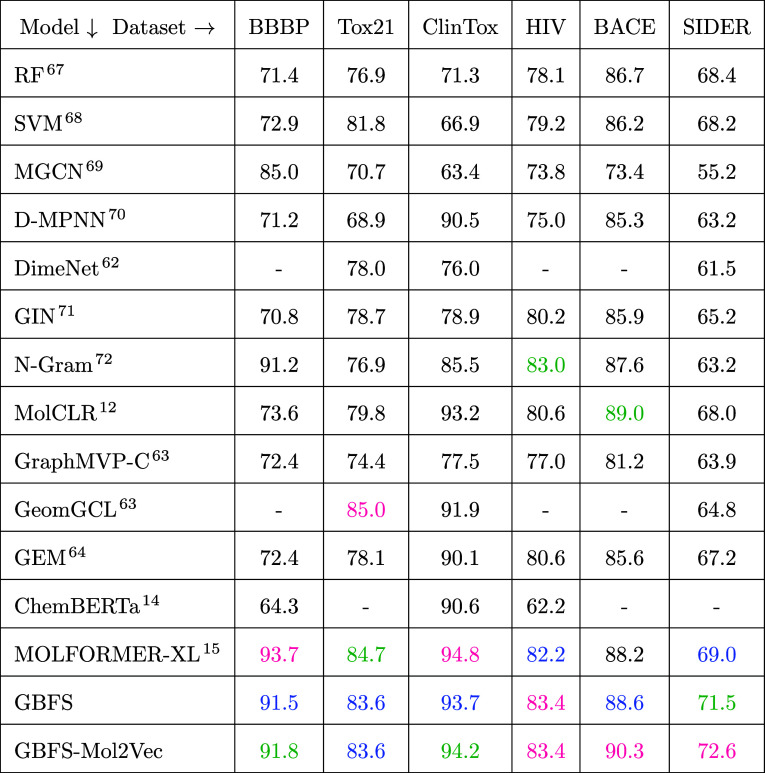
Model Performance on Classification Tasks^a^

a Model performance was assessed using AUC-ROC on scaffold splits, with color-coding indicating the top-performing model in pink, second-best in green, and third-best in blue. Performance benchmarks were obtained from multiple sources^12,14,15,63^

In the evaluation of these classification tasks, we included random forest (RF)^67^ and support vector machine (SVM)^68^ classifiers trained on molecular fingerprints, in addition to the previously mentioned ML models, as well as other graph-based networks and self-supervised models. These include: a multilevel graph convolutional network (MGCN)^69^ that is a neural-network architecture designed to process molecular graphs, preserving their intrinsic structure, and it uses a hierarchical graph-neural network to extract features directly from the spatial and conformational information, followed by multilevel interactions; directed-MPNN (D-MPNN),^70^ which differs from the generic MPNN, by using messages that are associated with directed edges instead of messages linked to vertices; a graph-isomorphism network (GIN)^71^ which is a graph-neural network pretrained on molecular graphs with edge features that uses a multilayer perceptron and a weighted sum of node features; the molecular contrastive learning representation (MolCLR)^12^ which is a self-supervised learning framework based on GIN with contrastive loss; N-Gram^72^ that employs an unsupervised approach for molecular representation, embedding nodes in a graph and constructing a representation by assembling the vertex embeddings in short graph walks; and GraphMVP-C, the graph multiview pretraining framework,^63^ which is a self-supervised learning framework that leverages the relationship between 2D topological structures and 3D geometry views.

The AUC-ROC analysis reveals interesting insights into performance variation between different models across a range of classification tasks. Among the existing supervised and semisupervised ML models (i.e., excluding our GBFS and GBFS-Mol2Vec workflows), RF and SVM models trained on molecular fingerprints demonstrate moderate performance across most tasks, with varying degrees of accuracy. They scored relatively high AUC-ROC scores on data sets such as Tox21, HIV, BACE and SIDER compared to MGCN, D-MPNN, and DimeNet. This performance is achieved in these models despite their lack of awareness regarding 3D molecular geometric information. Additionally, we observe that the semisupervised MGCN model and the supervised D-MPNN model outperform RF, SVM and the supervised DimeNet models on BBBP and ClinTox data sets; while these models generally yield satisfactory results, they may fall short in terms of the robustness required for precise predictions across all tasks.

We note that existing self-supervised or unsupervised ML methodologies, including pretrained molecular language models, generally outperform the aforementioned supervised ML models, exhibiting higher AUC-ROC scores across all classification tasks. For example, the MOLFORMER-XL model consistently ranks in the top three for AUC-ROC scores in five out of six classification tasks. Additionally, there are notable performances that surpass the MOLFORMER-XL model. The N-Gram model achieves one of the highest AUC-ROC scores on the HIV data set, while the MolCLR model records an AUC-ROC of 89.0 on the BACE data set, and the geometry-aware GeomGCL model attains the highest AUC-ROC score on the Tox21 data set. These scores underline their efficacy in capturing intricate molecular and drug properties across various data sets. In particular, the MOLFORMER-XL model excelled in achieving exceptionally high AUC-ROC values across all tasks, highlighting its strength and effectiveness in prediction across a variety of data sets.

We now introduce GBFS and GBFS-Mol2Vec into this comparative analysis and find that they emerge as top-performing ML models, demonstrating high performance across all classification tasks. Our models showcase superior predictive capability, particularly evident in classification tasks that are related to HIV, BACE and SIDER data sets. This highlights their versatility in capturing complex chemical-structural relationships with the target classes. Notably, both GBFS-based models consistently rank among the top-three performers across all data sets, as indicated by the color-coded results in Table 6. While graph-based models such as GraphMVP-C, GeomGCL, and GEM show promising performance in specific tasks, they may not consistently demonstrate the same level of robustness across all data sets when compared to the MOLFORMER-XL, GBFS, and GBFS-Mol2Vec models.

In summary, the results of this analysis emphasize the importance of model selection in classification tasks. They also demonstrate the effectiveness of sophisticated molecular language models, such as MOLFORMER-XL, alongside supervised or semisupervised methods, such as our GBFS and GBFS-Mol2Vec workflows, in capturing intricate chemical-structural characteristics across diverse data sets. These findings have significant implications for drug discovery and toxicity assessment in pharmaceutical research and development, thereby paving the way for diverse modeling strategies.

### 3.2 Case Study on Lipophilicity

Having assessed the performance of our GBFS and GBFS-Mol2Vec workflows across a diverse set of properties as detailed in Section 3.1, we conducted a case study to demonstrate the utility of our GBFS-based models in addressing the data-driven prediction of lipophilicity of chemical molecules. This also provides a good opportunity to exemplify the implementation of each stage of our GBFS-based operational workflows. Lipophilicity was selected for this case study because it is a measure of the ability of a molecule to dissolve in lipids, fats, oils or nonpolar solvents. This molecular attribute is important in drug discovery because it influences the extent to which the human body can uptake or metabolize a drug molecule.

Specifically, the octanol–water partition coefficient (*K*_ow_), or its logarithmic form (log *P*), quantifies the ratio of a solute’s concentration in a water-saturated octanolic phase to its concentration in an octanol-saturated aqueous phase, within a two-phase octanol–water system at equilibrium. This dimensionless measure serves as a common indicator of a chemical compound’s lipophilicity in drug discovery. Octanol serves as a model solvent because it mimicks lipid tissues in organisms and humans, as well as organic carbon in soils and sediments. This characteristic makes it a widely accepted surrogate for assessing chemical partitioning between aqueous and organic media. *K*_ow_ values range from 10^–3^ to 10^7^, corresponding to log *P* values that span from −3 to 7. These values indicate the propensity of a compound to partition itself between organic and aqueous phases. Lower log *P* values suggest higher water solubility and greater hydrophilicity, whereas higher log *P* values denote increased hydrophobicity.

A closely related concept is the octanol–water distribution coefficient (*D*_ow_), or its logarithmic form (log *D*). The two coefficients differ in that *D*_ow_ is pH-dependent, whereas *K*_ow_ is independent of a pH value. Chemical compounds can undergo partial ionization when dissolved in water, existing as weak acids or bases. Their pH value influences the proportion between the molecular and ionized forms of a drug. For ionizable solutes, *D*_ow_ serves as a measure of the pH-dependent differential solubility across all species, accounting for both neutral and ionized concentrations within the octanol–water system. The extent to which an ionizable compound dissociates across environmentally relevant pH ranges can greatly affect properties such as water solubility. Therefore, a pH-dependent estimate of lipophilicity is a crucial attribute of drug molecules, exerting influence on both membrane permeability and solubility. In this case study, we trained GBFS and GBFSMol2Vec models to predict the lipophilicity of chemical molecules, leveraging experimental data on the octanol–water distribution coefficient (log *D* at pH 7.4).

We note that the two GBFS-based models remain indistinguishable until the optimization stage of the GBFS-based workflow. This is because the substructure vector embeddings that handle molecular representations are integrated into the final subset of features which are selected by the GBFS workflow just before this optimization step. Consequently, the results presented in the subsequent section are applicable to both workflows.

#### 3.2.1 Performance Results

**Figure 2:**
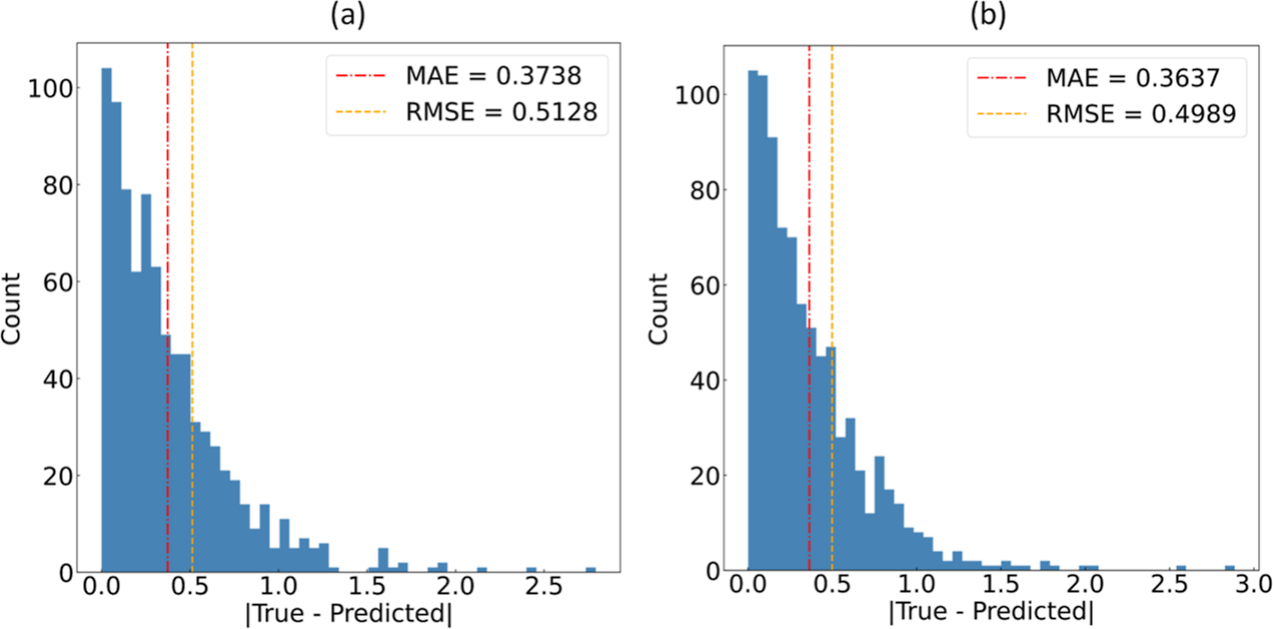
Distribution plot of the absolute errors resulting from the ML predictions of lipophilicity in chemical molecules within the test set, realized through the (a) GBFS and (b) GBFS-Mol2Vec workflows, respectively. The red dot-dashed line (-.) signifies the MAE, while the orange dashed line (--) represents the RMSE.

The regression analysis of log *D* was performed using a gradient-boosting algorithm, employing 129 features selected via our GBFS-based workflow from a pool of ca. 11,000 features. The outcomes have already been detailed in Table 4, showing that GBFS and GBFS-Mol2Vec models afforded the lowest RMSE. The corresponding error distributions are depicted in Figure 2. The use of our GBFS workflow afforded MAE and RMSE values of 0.3738 and 0.5128, respectively, while the GBFS-Mol2Vec approach yielded MAE and RMSE values of 0.3637 and 0.4989, respectively. The slightly higher error in RMSE values is due to the increased penalization of predictions that show larger deviations from their true values, as per eqs 1 and 2. The performance of both of our GBFS-based models surpasses that of alternative methods, as evidenced by the range of RMSE values detailed in Table 4.

#### 3.2.2 GBFS Process

**Figure 3:**
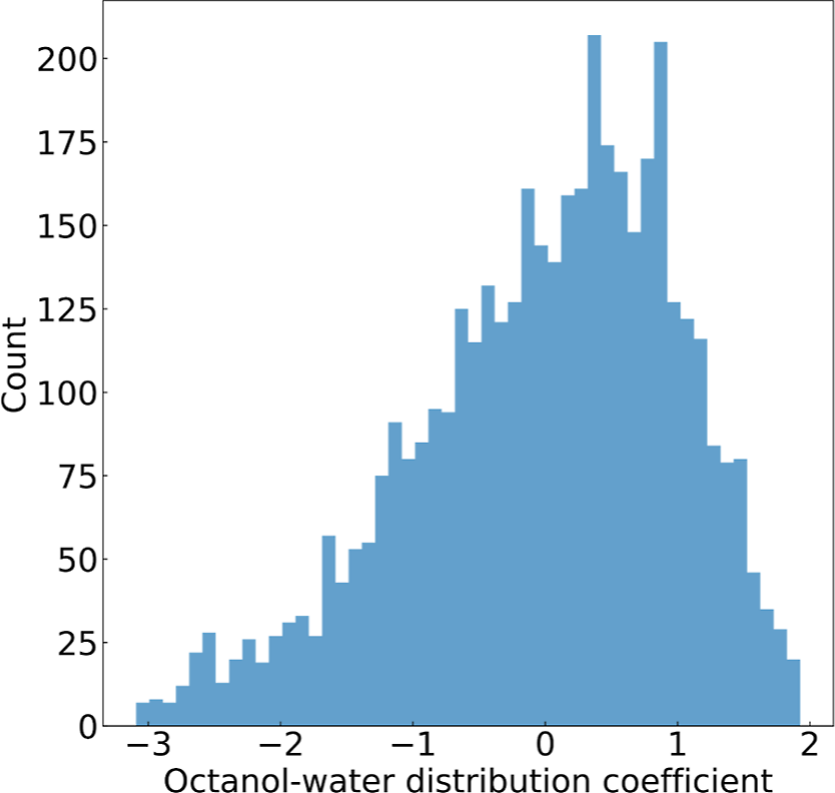
Distribution of experimental octanol–water distribution coefficient (log *D*) values in the lipophilicity data set.

The regression analysis involved 4200 chemical compounds, partitioned with a train-to-test ratio of 4:1. Figure 3 illustrates the distribution plot of log *D* values within the data set, ranging from −3.09 to 1.93. Unlike some of the aforementioned studies that employed other ML models using a train-to-test split ratio of 9:1, we intentionally opted for a smaller training set. We exclusively trained our GBFS-based models on SMILES-based features, adhering to a more rigorous and stringent criterion for model assessment.

**Figure 4:**
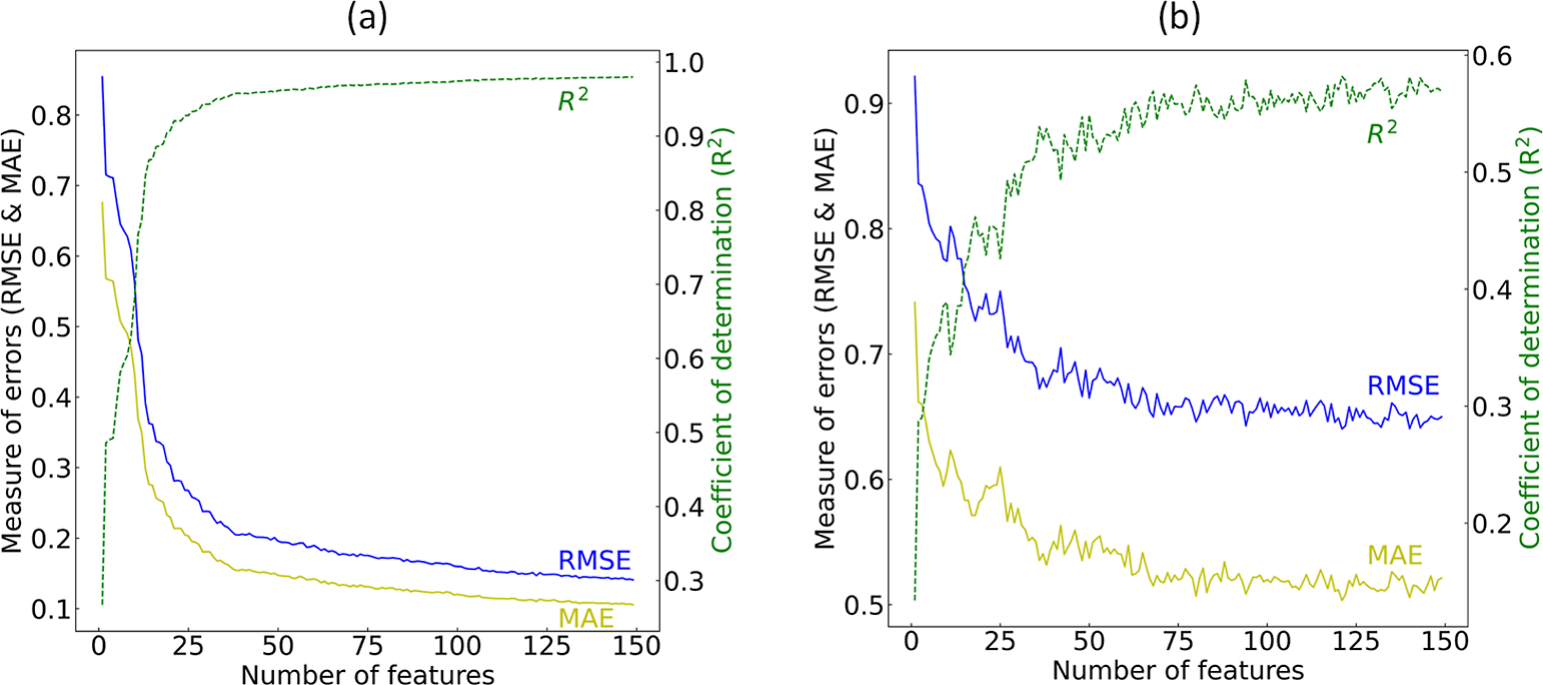
Result of the GBFS process in the regression analysis of log *D*. Model performance of the GBDTs is depicted separately for (a) the training set and (b) the validation set. Regression models are recursively trained with an incrementally increasing subset of features, starting from the most relevant feature as determined by the total loss reduction achieved.

The recursive training of GBDTs with an increasing subset of features demonstrated convergence of MAE, RMSE and *R*^2^ statistical figures-of-merit on the training set using ca. 75 of the most relevant features. An incremental improvement in the errors was observed for up to ca. 150 features. The ranking of feature relevance was determined by calculating the extent of loss reduction, or variance gain for each feature during the training of a GBDT using the entire set of features. Similarly, on the validation set, all four performance statistics reached a plateau before the first ca. 75 features were incorporated during the model training. As they converged, the validation set exhibited poorer performance with more noise, in line with expectations for an out-of-sample set. The performance of the regression models during the feature-selection process, evaluated on both the training and validation sets, is depicted in Figure 4.

#### 3.2.3 Feature Analyzes & Feature Engineering

Concurrently, we implemented bivariate hypothesis testing methods, primarily to understand the causal relationships between exploratory features and the target variable. This analytical approach is aimed at identifying direct influences that individual features may have on the property prediction. For instance, a comparison of means was conducted using the F-test in one-way analysis of variance (ANOVA). This analysis involves performing correlation analysis using the Pearson correlation coefficient (*R*) for two continuous features. The analysis then progresses to an ANOVA-based regression approach, wherein *R* is transformed into a regression *F*-statistic to quantify the strength of the relationship. In other words, a hypothesis-based testing method was employed for statistical inference, with the significance of a feature being inferred from the test statistics that are generated by testing the hypothesis that their exists an association between two features.

Additionally, mutual information (MI) analysis was conducted to further explore interfeature dependencies. This analysis quantifies the mutual dependence between pairs of features by measuring the information gain or entropy observed through one feature about another. MI evaluates the disparity between the joint distribution of a feature pair and the product of their marginal distributions. A higher MI value signifies a stronger dependency between the features. Herein, we adopted an MI estimator that is based on entropy estimations that are derived from *k*-nearest neighbor distances.

We assessed the linear association of each continuous exploratory feature with the target variable through a normalized F-statistic for relative comparison. Thereby, we found that the feature demonstrating the highest linear association with the target variable was Crippen–Wildman partition coefficient (MolLogP) estimate from an atom-based scheme using literature values.^74^ This top-ranking feature was closely followed by those concerning the minimum partial charge (MinPartialCharge), the number of carboxylic acids (fr-COO) and the number of aromatic hydroxyl groups (fr-Ar-OH), with normalized F-statistics scoring above 0.68.

The MI analysis indicated that the greatest amount of entropy gain was realized when considering the Crippen–Wildman partition coefficient estimate, closely followed by the minimum partial charge and the maximum absolute value of the electrotopological-state index (MaxAbsEStateIndex), with a normalized MI score of 0.999 and 0.928, respectively. In essence, the MI analysis, incorporating the *k*-nearest neighbors method, suggests that more accurate predictions of log *D* can be achieved by considering the Crippen–Wildman partition coefficient estimate that can be derived from atom-based calculations. The estimation of MI involves assessing the probability density and marginal distributions of two variables. However, this task becomes increasingly complex in high-dimensional data owing to the disproportionate ratio of dimensions to samples. This disparity often results in significant variations in probability estimates which consequently affect the reliability of the inferred information gain in MI analysis. Such issues stem from the high dimensionality of the data set or insufficient sample density relative to the dimension of the feature space.

The features identified through our initial calculation of the loss reduction and statistical analyses were used to engineer new features via the brute-force method. This process resulted in an additional 42 features, bringing the total number of features to 192, which formed the preliminary subset for the regression analysis.

#### 3.2.4 Multicollinearity Reduction, Permutation Analysis & Recursive Feature Elimination

In the subsequent phase of the GBFS-based workflow, we address multicollinearity reduction within the lipophilicity data set, assess the permutation importance of the selected features, and conduct RFE to ascertain the final subset of features for Bayesian optimization of the final predictive ML model.

**Figure 5:**
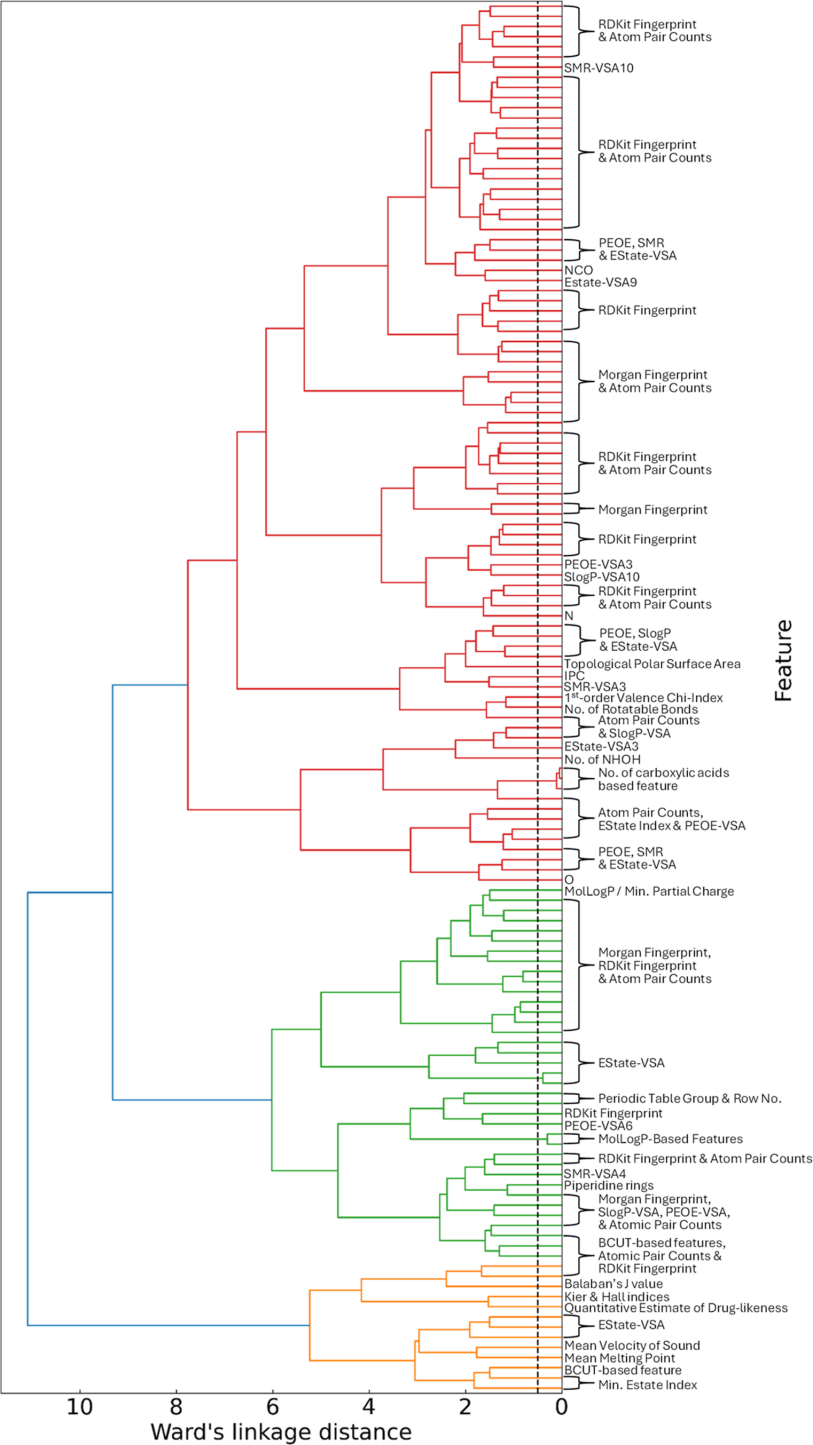
Dendrogram of the hierarchical agglomerative clustering. The dashed line in black represents the threshold of 0.5 units of Ward’s linkage distance. The dendrogram has been rotated to improve the visibility of the labels.

To mitigate the effects of multicollinearity in the lipophilicity data set, features with a correlation coefficient of 0.8 or higher were systematically removed. The next remediation of multicollinearity effects involved employing a hierarchical cluster analysis, using Spearman rank-order correlation with a Ward’s linkage distance threshold of 0.5 units. This led to the retention of 133 features. The dendrogram depicted in Figure 5 illustrates hierarchical agglomerative clustering and cluster formation as one ascends.

**Figure 6:**
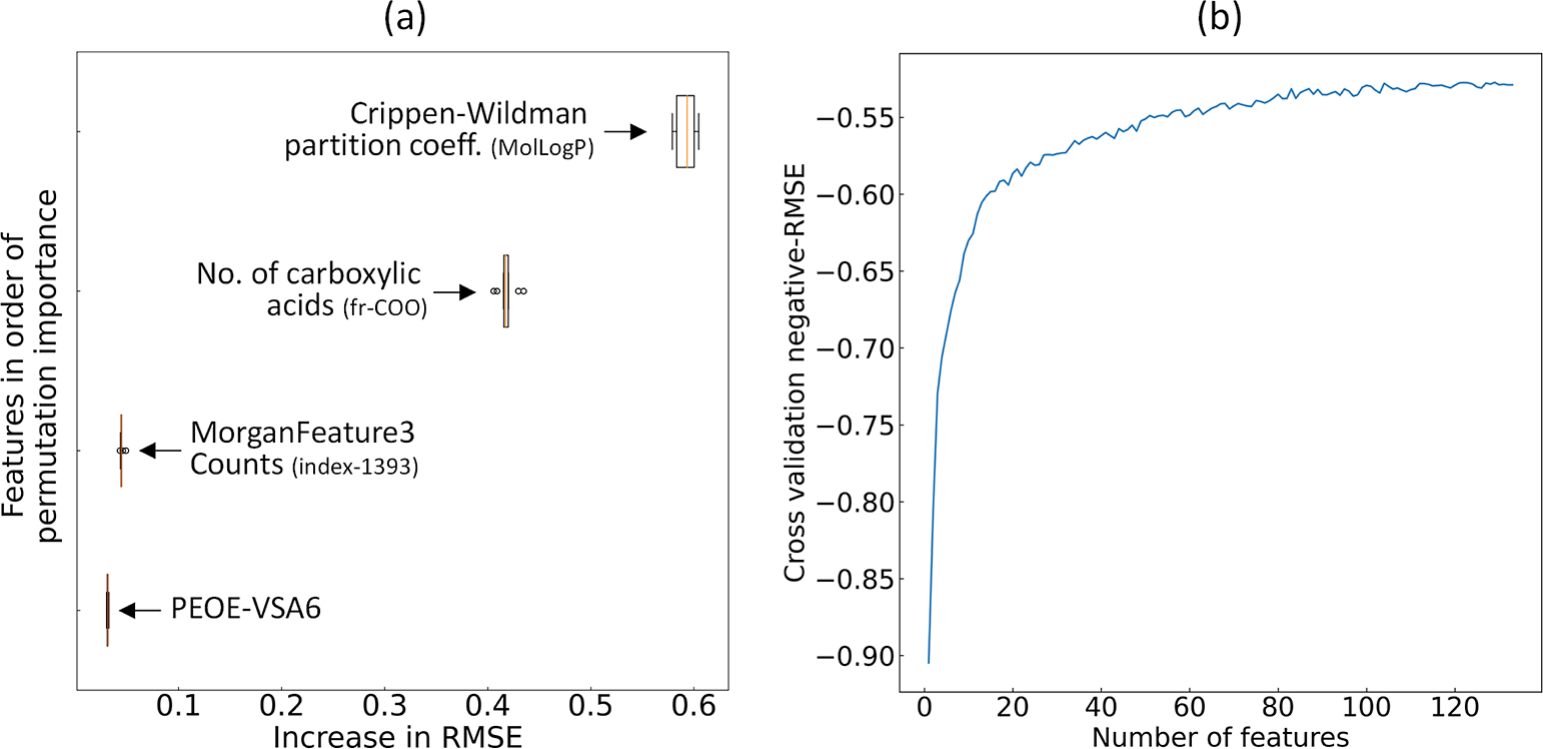
(a) Permutation feature-importance plot for the regression analysis of lipophilicity, and (b) 10-fold RFE result using RMSE as the performance metric.

Permutation feature importance is quantified as the reduction in model performance when a single feature is randomly shuffled. This process disrupts the association between the feature and the target, making the reduction in model performance indicative of the model’s reliance on that particular feature. The result of the 10-fold permutation feature-importance analysis is shown in Figure 6a. The 10-fold feature permutation analysis shows that the most important feature is the Crippen–Wildman partition coefficient estimate, followed by the number of carboxylic acids. The results are consistent with the statistical analyses that were conducted independently.

Subsequently, the optimal subset of features was determined by eliminating further features through 10-fold RFE, using negative RMSE as the performance metric; Figure 6b displays the cross-validation result. RFE follows a greedy-based search strategy, recursively training an estimator with varying combinations of features. This process continues until the most compact subset of features that yields optimal performance is identified. This process led to final subset being identified which comprises 129 features, chosen from an initial pool of more than 11,000 base features and 42 engineered features. These features showed the highest relevance to the target variable without any prior knowledge of the domain.

#### 3.2.5 Model Optimization

A two-step optimization process was followed to determine the architecture of each final GBFS-based model. Initially, a grid search was performed to scan a wide hyperparameter space. This step helped to identify a promising region within the hyperparameter space, where Bayesian optimization was subsequently applied to fine-tune more selective hyperparameters precisely. Such an optimization strategy proves to be particularly effective for an objective function that has no closed form, is expensive to evaluate, and when the evaluations are noisy. The convergence, partial dependence and evaluation plots from Bayesian optimization of the GBFS-based model are shown in Supporting Information 1, along with the pseudocode for Bayesian optimization in Supporting Information 2.

**Figure 7:**
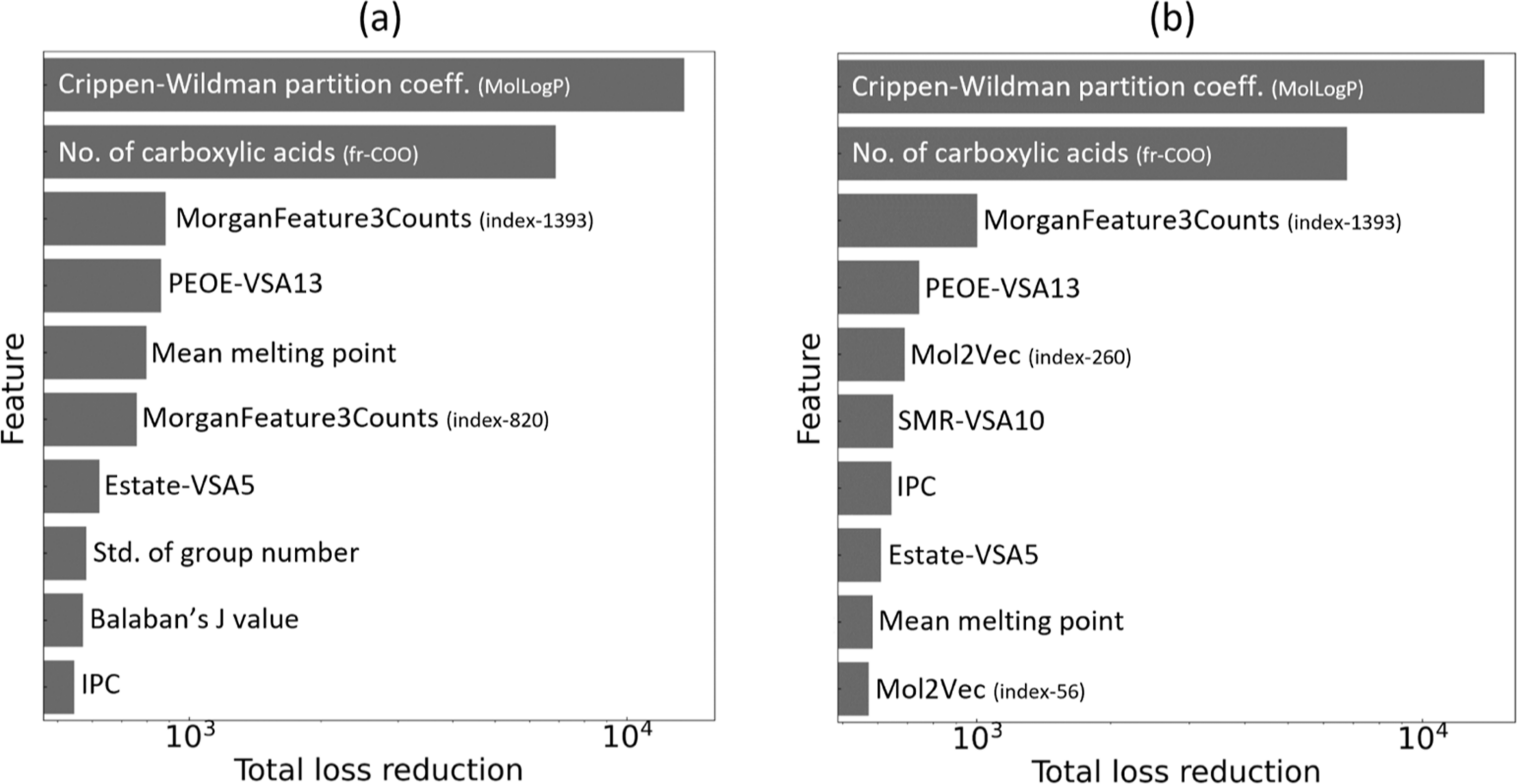
Feature-importance result showing the final 10 most relevant features that were selected for the regression analysis of lipophilicity and the realized total loss reduction via the (a) GBFS and (b) GBFS-Mol2Vec workflow, respectively.

As previously noted, the results presented thus far in our GBFS-based workflow are identical for both the GBFS and GBFS-Mol2Vec models. Following this stage, the procedure for the GBFS-Mol2Vec model differs from that of the GBFS workflow. Thereby, the GBFS-Mol2Vec workflow integrates the features derived from Mol2Vec into the GBFS workflow during its optimization phase. The total loss reduction (i.e., the feature-relevance ranking) realized by the final set of features is depicted in Figure 7a for the GBFS model and Figure 7b for the GBFS-Mol2Vec model. The descriptions of the final features used to train the GBFS-Mol2Vec model, which afforded the lowest error, are provided in Table 7; the final features selected by the GBFS model are similar, except of course for the lack of any Mol2Vec-based features.

**Table 7:** Features That are Identified to Have the Most Relevance in the Prediction of Lipophilicity, Listed in the Order of Importance (with One Being the Highest)

no.	feature abbreviation	feature description
1	MolLogP	Crippen–Wildman partition coefficient estimate from an atom-based scheme using values in ref (74)
2	fr-COO	number of carboxylic acids
3	fm3-1393	count-based Morgan fingerprint features of radius 3 (index 1393)
4	PEOE-VSA13	Molecular-Operating-Environment (MOE)-type descriptors using partial charges and van der Waals surface area contributions (index 13)
5	Mol2Vec-260	molecular-to-vector (index 260)
6	SMR-VSA10	MOE-type descriptors using molar refractivity and van der Waals surface area contributions (index 10)
7	IPC	information content of the coefficients of the characteristic polynomial of the adjacency matrix of a hydrogen-suppressed graph of a molecule based on ref (76)
8	EState-VSA5	MOE-type descriptors using electrotopological state indices and van der Waals surface area contributions (index 5)
9	mean melting point	mean melting point estimated using Pymatgen data^50^
10	Mol2Vec-56	molecular-to-vector (index 56)
11	fm3-820	count-based Morgan fingerprint features of radius 3 (index 820)
12	Std. dev. group	standard deviation of group number
13	BalabanJ	Balaban’s *J* value for a molecule based on ref (77)

#### 3.2.6 SHAP Analysis

**Figure 8:**
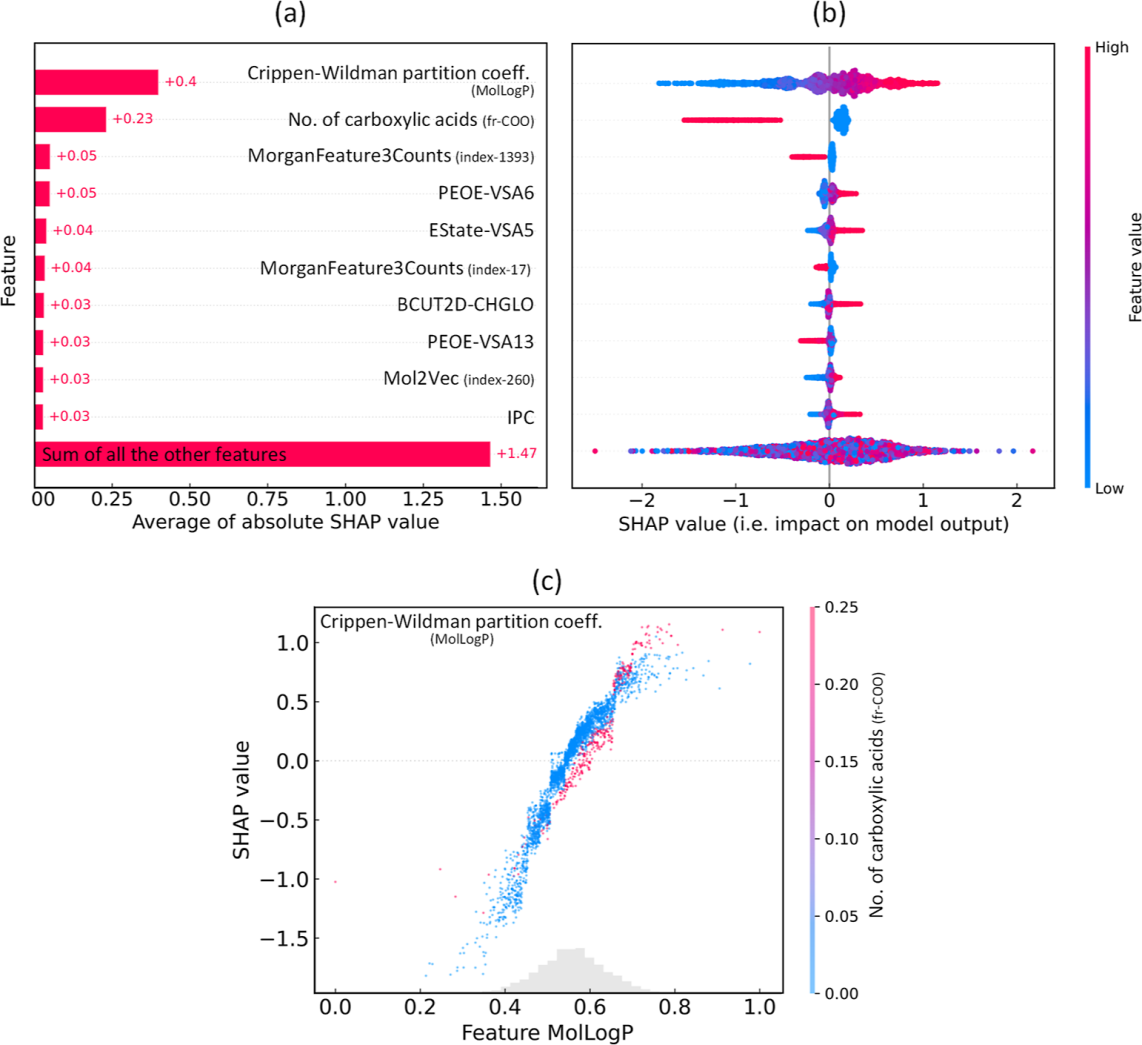
Results based on the SHAP framework: (a) the average contribution (i.e., the mean absolute SHAP value) of ten features that are identified as having the most contribution to the GBFS-Mol2Vec model output. (b) The beeswarm plot illustrates the impact of these features on the GBFS-Mol2Vec model output by plotting each instance as a single data point together with the SHAP value on the *x*-axis, where the *y*-axis is consistent with (a). A positive SHAP value indicates a positive contribution to the prediction of lipophilicity. The color scheme corresponds to the original feature value, while the broadening shows the density of instances (cf. density plot). (c) The SHAP distribution of Crippen–Wildman partition coefficient estimates (MolLogP) as a feature, and the data distribution as a gray histogram along the *x*-axis. The color scheme corresponds to the feature value of the number of carboxylic acids (fr-COO).

An independent feature analysis was conducted using the SHapley Additive exPlanations (SHAP) framework,^75^ which is a game theoretic approach to explain the output of an ML model. Figure 8a displays the average contribution plot (i.e., the mean absolute SHAP value) of ten features identified as having the most significant contribution to the output of the GBFS-Mol2Vec model. The accompanying beeswarm plot in Figure 8b shows the influence of features on the output of the GBFS-Mol2Vec model by plotting each instance as a single data point together with the SHAP value on the *x*-axis. Moreover, Figure 8c depicts the Crippen–Wildman partition coefficient estimates as a feature, and the data distribution as a gray histogram along the *x*-axis. The color scheme corresponds to the feature value corresponding to the number of carboxylic acids. Once again, the most significant feature was identified as the Crippen–Wildman partition coefficient estimate, followed closely by the feature pertaining to the number of carboxylic acids. These findings support the results of the statistical analyses and align with those identified through our GBFS-based workflows, providing additional confirmation of the efficacy of our modeling methodology.

#### 3.2.7 Feature Interpretation

We have presented a methodical modeling strategy for material-property predictions with promising statistical figures-of-merit, focusing on the lipophilicity of chemical compounds as an illustrative case. Nevertheless, it is imperative to delve into feature interactions and examine the physical significance of these features in relation to our final predictions.

We began our case study by introducing the concept of the octanol–water partition coefficient, which differs from the octanol–water distribution coefficient, as it is a pH-independent measure of lipophilicity. This coefficient, denoted as log *P*, can be derived from molecular structures instead of being determined experimentally. In this case study, we computed the Crippen–Wildman partition coefficient using an atom-based scheme, which employs lipophilicity data from Wildman and Crippen.^74^ Since log *P* also directly measures lipophilicity, it is unsurprising that the inclusion of the Crippen–Wildman log *P* among the final subset of features proved to be most relevant for predicting log *D*.

Distinct patterns emerge from a thorough examination of the relationship between Crippen–Wildman log *P* and experimental log *D* values within the training data set. The correlation coefficient between these two variables is ca. 0.42, indicating a noticeable positive trend where higher log *P* values tend to correlate with higher log *D* values, both reflecting increased lipophilicity. This observation finds further support in Figure 8b,c, where larger Crippen–Wildman log *P* values generally align with more positive SHAP values. Notably, the threshold at which Crippen–Wildman log *P* exhibits a negative SHAP value appears to be below ca. 0.5.

Moreover, a clear trend is observed between the number of carboxylic acids and the log *D* values, showing that a higher number of carboxylic acids correspond to lower log *D* values. Its correlation with the log *D* values in the training data is not as pronounced as that of Crippen–Wildman log *P*, registering a correlation of ca. −0.33. This indicates that a lower number of carboxylic acids tends to exhibit larger log *D* values. Such a pattern is observable in Figure 8b,c, where there is distinct segregation between the positive and negative SHAP values, corresponding to a lower and higher number of carboxylic acids.

This observation aligns with fundamental principles of chemistry. Carboxylic acids are polar because their structures allow them to serve as both hydrogen-bond acceptors (via the carbonyl –C(=O)−) and hydrogen-bond donors (through the hydroxyl –OH). In nonpolar environments, carboxylic acids tend to self-associate through hydrogen bonding among their acidic molecules, resulting in the formation of dimers. This increases the effective size of the molecules, thus enhancing the van der Waals dispersion forces between these dimers and adjacent molecules. Consequently, when compared to other organic compounds of similar molecular weight, this characteristic contributes to high boiling and melting points, the molecular attribute that emerged as the fifth most relevant feature influencing the prediction of the target variable, log *D*, in our results pertaining to the GBFS model (see Figure 7a).

In an aqueous environment, carboxylic acids do not undergo dimerization; instead, their individual acidic molecules form hydrogen bonds with water molecules. Carboxylic acids with alkyl chains containing one to four carbon atoms are soluble in water. As the length of the alkyl chain increases, solubility diminishes rapidly due to the increasing hydrophobicity of the alkyl moiety. The elongated hydrocarbon tails of these molecules have a tendency to disrupt hydrogen bonds among water molecules, which are subsequently substituted by weaker van der Waals dispersion forces. For instance, palmitic acid (COOH), as characterized by its extensive nonpolar hydrocarbon chains, is insoluble in water. In comparison, hexanoic acid (COOH), containing shorter nonpolar hydrocarbon chains, exhibits minimal solubility in water. Carboxylic acids typically demonstrate solubility in organic solvents like ethanol, toluene, and diethyl ether. This fundamental chemistry underscores the significance of the number of carboxylic acids, which is ranked second in Table 7. It is worth emphasizing that the total loss reduction during the training process for both GBFS-based models was predominantly driven by two features: Crippen–Wildman log *P* and the number of carboxylic acids. This significant contribution is clearly shown in Figure 7a,b.

A modest, yet non-negligible correlation exists between the log *D* of a molecule and its van der Waals dispersion forces. Among the feature attributes outlined in Table 7, it is evident that three features associated with van der Waals surface area (VSA) contributions were identified by the GBFS and GBFS-Mol2Vec workflows: PEOE-VSA13, SMR-VSA10, and EState-VSA5, registering correlations of −0.11, 0.11 and 0.19, respectively. These descriptors belong to a hybrid, molecular-operating-environment (MOE) category, wherein the contribution of each atom in the molecule to a molecular property is computed alongside their VSA contribution. Subsequently, the atoms are categorized into bins based on the property contributions, followed by the summation of the VSA contributions for each atom within a bin. The index number of these features corresponds to the bin number.

Furthermore, two Mol2Vec embeddings Mol2Vec-260 and Mol2Vec-56 ranked fifth and tenth in importance in the feature selection shown in Table 7, which corresponds to the GBFS-Mol2Vec model. While the impact of these features on the total loss reduction is understandably less pronounced than that of the Crippen–Wildman log *P* estimates and the number of carboxylic acids, their inclusion resulted in a lower RMSE value on the same test set. Although an interpretation of the individual Mol2Vec embeddings can be challenging, previous studies have demonstrated their utility in transfer learning. Specifically, these embeddings of substructure vectors, acquired through unsupervised learning on unlabeled chemical compounds, can enhance material-property models with limited data sets through transfer learning. In this case study, we leveraged the acquired embeddings to enhance the prediction of lipophilicity.

#### 3.2.8 Out-Of-Sample Prediction Examples

The results discussed thus far present predictions of log *D* values against experimental measurements, with promising statistical figures-of-merit. Nonetheless, it is important to validate these results by considering how these predictions fare against the diverse range of chemical molecules rather than simply demonstrating their collective statistical quality in an anonymized form. A further comparative analysis was, therefore, conducted against chemical molecules that have not been previously seen by the model. The corresponding out-of-sample predictions of log *D* were subsequently compared against reference experimental measurements, with results from 60 randomly selected examples being summarized in Table 8.

**Table 8:** Examples of Input SMILES Strings and the Corresponding Prediction of Lipophilicity (log *D*), Sorted by the Absolute Percentage Difference from Experimentally Measured Literature Values^28−30^ in Ascending Order

	prediction input		prediction output		
chemical formula	SMILES	expt	pred	pred.–expt	% diff
C_8_H_16_N_4_S	CC(C)N(C(C)C)c1nnc(N)s1	–0.59065	–0.58704	0.00361	0.6
C_19_H_23_FN_4_O_2_	Cc1ccc(COC(=O)N2CC[C@H](CNc3ncccn3) [C@H](F)C2)cc1	0.84776	0.85311	0.00535	0.6
C_9_H_13_N_3_O	CC(C)NNC(=O)c1ccncc1	–1.56074	–1.54842	0.01233	0.8
C_18_H_16_FNO_6_S_2_	Cc1c(CC(=O)O)c2cc(F)ccc2n1S(=O)(=O)c3ccc(cc3)S(=O)(=O)C	–2.20469	–2.22211	–0.01743	–0.8
C_28_H_26_F_3_N_3_O_7_	CC(C)C(NC(=O)CN1C(=O)C(=CC=C1c2ccccc2)NC(=O)OCc3ccc(cc3)C(=O)O)C(=O)C(F)(F)F	–1.13424	–1.12462	0.00961	0.8
C_26_H_23_N_5_O	C[C@@H](NC1=CC(=O)CC1)c2ccc(Nc3ncc4cc(ccc4n3)c5ccncc5)cc2	0.83940	0.83187	–0.00753	–0.9
C_15_H_18_N_4_O_2_S_2_	NC(=O)Nc1sc(cc1C(=O)N[C@H]2CCCNC2) c3ccsc3	–0.95025	–0.95886	–0.00861	–0.9
C_21_H_18_ClN_5_O_4_	CN(C)C(=O)Nc1ccc(CN2NC(=O)C3=C(C2=O$C(=O)c4ccc(Cl)cc4N3)cc1	–0.91680	–0.90843	0.00837	0.9
C_25_H_26_ClN_7_O_3_	COc1cc(ccc1Nc2ncc(Cl)c(n2)c3cnc4cc(CO)ccn34)N5CCN(CC5)C(=O)C	0.75577	0.76341	0.00764	1.0
C_10_H_14_N_2_	CN1CCCC1c2cccnc2	–1.41858	–1.43342	–0.01485	–1.0
C_15_H_22_FN_3_O_4_S	CC(C)(CS(=O)(=O)N1CCN(CC1)c2ccc(F)cc2)N(O)C=O	–0.94189	–0.95268	–0.01079	–1.1
C_24_H_29_F_3_N_2_ O_3_S	CCN(C1CCN(Cc2ccc(cc2)C(F)(F)F)CC1)C(=O)Cc3ccc(cc3)S(=O)(=O)C	0.58852	0.58044	–0.00807	–1.4
C_23_H_29_N_3_O_5_S	CN(CCNC[C@H](O)c1ccc(O)c2NC(=O)Sc12)C(=O)CCOCCc3ccccc3	–0.82481	–0.83644	–0.01162	–1.4
C_25_H_28_F_3_N_3_O_2_	COc1ccc2c(C)cc(N[C@H]3CCC[C@@H](C3)NCc4cccc(OC(F)(F)F)c4)nc2c1	1.18228	1.16486	–0.01742	–1.5
C_24_H_29_Cl_2_N_3_ O_5_S	COc1ccc(cc1)S(=O)(=O)NC(=O)N2CCC(CC2)N3CCC(CC3)Oc4ccc(Cl)c(Cl)c4	–0.67428	–0.68564	–0.01136	–1.7
C_25_H_30_N_6_O_2_	CN(c1ccccc1)c2ccnc(Nc3cc(cc(c3)N4CCOCC4)N5CCOCC5)n2	1.06520	1.04686	–0.01834	–1.7
C_15_H_17_F_4_N_5_O_4_	C[C@H]1O[C@H]([C@H](O)[C@@H]1O)n2cnc3c(N)nc(OCC4CC(F)(F)C4(F)F)nc23	–0.64919	–0.63778	0.01141	1.8
C_24_H_23_Cl_2_N_3_O_4_	OC(=O)[C@H](Cc1cccc(OCCCNc2ccccn2)c1)NC(=O)c3c(Cl)cccc3Cl	–1.70291	–1.73467	–0.03176	–1.9
C_22_H_23_F_5_N_6_ O_3_S	OC[C@H]1C[C@H]([C@H](O)[C@@H]1O)n2nnc3c(N[C@@H]4C[C@H]4c5ccc(F)c(F)c5)nc(SCCC(F)(F)F)nc23	1.50007	1.53064	0.03057	2.0
C_18_H_16_ClNO_4_S_2_	Cc1c(Sc2ccc(Cl)cc2)c3cc(ccc3n1CC(=O)O)S(=O)(=O)C	–1.38512	–1.35537	0.02975	2.1
C_25_H_27_N_5_O_4_	O[C@@H]1CN(CCN2C(=O)C = Cc3ccc(cc23)C#N)CC[C@@H]1NCc4cc5OCCOc5cn4	–1.45203	–1.41925	0.03277	2.3
C_8_H_8_N_4_O	NNC1=Nc2ccccc2NC1=O	–1.46875	–1.43553	0.03323	2.3
C_24_H_25_N_3_O_3_	COc1ccc(cc1)C(=O)Nc2cc(NC(=O)c3cccc(c3)N(C)C)ccc2C	0.81431	0.79583	–0.01849	–2.3
C_25_H_31_N_5_O_3_	C[C@@H]1CN(CCN1C(=O)[C@@H]2CCCC [C@H]2C(=O)NC3(CC3)C#N)c4ccc5c(C)noc5c4	0.39617	0.38714	–0.00902	–2.3
C_26_H_23_F_2_N_3_O_3_	C[C@H](NC(=O)Cc1cc(F)cc(F)c1)C$=O)NC2C(=O)N(C)c3ccccc3c4ccccc24	1.17392	1.14022	–0.03370	–2.9
C_22_H_27_ClN_2_O_5_S	C[C@H]1CN(Cc2cc(Cl)ccc2OC(C)(C)C(=O)O)CCN1S(=O)(=O)c3ccccc3	–1.38512	–1.42671	–0.04158	–3.0
C_16_H_13_F_3_O_4_	COc1ccc(cc1)c2cc(ccc2OCC(=O)O)C(F)(F)F	–1.59420	–1.64238	–0.04818	–3.0
C_13_H_18_ClNO_2_	C[C@@H]1NC(C)(C)CO[C@@]1(O)c2cccc(Cl)c2	–0.42339	–0.41045	0.01294	3.1
C_11_H_12_F_3_N_5_	FC(F)(F)c1nnc2ccc(nn12)N3CCCCC3	0.67214	0.64994	–0.02220	–3.3
C_19_H_15_ClN_4_O_3_	CCC(N1NC(=O)c2nc3cc(Cl)ccc3c(O)c2C1=O)c4cccnc4	–1.25132	–1.20065	0.05067	4.0
C_20_H_21_FN_2_O	CN(C)CCCC1(OCc2cc(ccc12)C#N)c3ccc(F)cc3	–0.53211	–0.55394	–0.02183	–4.1
C_20_H_19_ClN_4_O_5_	COc1cc(Nc2cc(Nc3c(Cl)ccc4OCOc34)ncn2)cc(OC)c1OC	0.73068	0.76155	0.03086	4.2
C_13_H_9_FN_2_	Fc1ccc(cc1)c2cn3ccccc3n2	0.75577	0.78804	0.03227	4.3
C_23_H_25_ClN_4_O_2_S	Cc1sc2c(C(=N[C@@H](CC(=O)OC(C)(C)C)c3nnc(C)n23)c4ccc(Cl)cc4)c1C	1.35790	1.41848	0.06058	4.5
C_15_H_16_Cl_2_N_4_ O_3_S	Cc1[nH]c(C(=O)NC2CCN(CC2)c3nc(cs3)C(=O)O)c(Cl)c1Cl	–1.71964	–1.80957	–0.08993	–5.2
C_6_H_11_NO	O=C1CCCCCN1	–1.90362	–2.00360	–0.09997	–5.3
C_25_H_24_F_3_N_3_O_5_	CC1=CN([C@H]2CCCN(Cc3ccc(C(=O)O)c(Oc4cccc(c4)C(F)(F)F)c3)C2)C(=O)NC1=O	–2.15451	–2.04005	0.11446	5.3
C_31_H_36_ClN_3_O_5_	CC1=CN([C@H]2CCCN(C2)[C@H](CC3CCCCC3)c4ccc(C(=O)O)c(Oc5cccc(Cl)c5)c4)C(=O)NC1=O	–0.30631	–0.29003	0.01628	5.3
C_23_H_25_BrN_4_O	CO[C@@H]1CC[C@@]2(CC1)Cc3ccc(cc3C24N=C(C)C(=N4)N)c5cncc(Br)c5	1.00666	0.95149	–0.05516	–5.5
C_11_H_20_N_2_O_2_	CC(C)(C)C(=O)N[C@H]1CCCCNC1=O	–1.35167	–1.26705	0.08462	6.3
C_26_H_24_N_2_O_3_S	CC[C@H](NC(=O)c1c(c(nc2ccccc12)c3ccccc3)S(=O)(=O)C)c4ccccc4	0.81431	0.86580	0.05149	6.3
C_17_H_17_N_3_O_2_	COc1cc2ncnc(NCc3ccccc3)c2cc1OC	0.91467	0.97275	0.05808	6.3
C_20_H_21_F_3_N_2_ O_5_S	OC(=O)COc1ccc(cc1CN2CCN(CC2)S(=O)(=O)c3ccccc3)C(F)(F)F	–1.50220	–1.59854	–0.09634	–6.4
C_15_H_11_FN_4_O_2_	OC(=O)c1ccc(cc1)c2nnn(Cc3ccccc3F)n2	–1.58583	–1.68813	–0.10230	–6.5
C_24_H_22_N_4_O_2_	COc1ccc(Nc2 cc(Oc3cc(C)c(C)nc3c4ccccn4)ccn2)cc1	1.09865	1.01593	–0.08272	–7.5
C_16_H_14_ClFO_5_S	CCS(=O)(=O)c1ccc(c(F)c1)c2cc(Cl) ccc2OCC(=O)O	–2.60610	–2.40706	0.19905	7.6
C_22_H_28_ClN_3_ O_3_S_2_	O[C@@H](CNCCCSCCNCCc1cccc(Cl)c1)c2ccc(O)c3NC(=O)Sc23	–0.95025	–1.02418	–0.07393	–7.8
C_21_H_25_N_3_O_2_S	O=S(=O)(NCC(N1CCCCCC1)c2ccccc2)c3ccc(cc3)C#N	0.55506	0.51115	–0.04392	–7.9
C_21_H_25_ClFN_7_O_2_	C[C@H](Nc1nc(NC[C@@H](O)CO)c(Cl)c(Nc2cc([nH]n2)C3CC3)n1)c4ccc(F)cc4	0.99830	0.91922	–0.07908	–7.9
C_16_H_18_N_2_	CN1C[C@@H](c2ccccc2)c3cccc(N)c3C1	–0.07215	–0.07852	–0.00637	–8.8
C_7_H_8_N_2_O	CC(=O)Nc1ccccn1	–1.41858	–1.54491	–0.12633	–8.9
C_25_H_29_F_3_N_2_ O_2_S_2_	CC(C)CN1C(=O)N(C)C(=O)c2c(SC3CC CCC3)c(Cc4ccccc4C(F)(F)F)sc12	1.62551	1.77093	0.14542	8.9
C_22_H_25_F_3_N_2_ O_4_S	CC(C)Oc1ccc(cc1C(=O)N2CCN(CC2) c3ccc(cc3)C(F)(F)F)S(=O)(=O)C	0.94812	1.03297	0.08485	8.9
C_20_H_15_F_3_N_4_O_3_	NC1C2CN(CC12)c3nc4N(C=C(C(=O)O)C(=O)c4cc3F)c5ccc(F)cc5F	–1.58583	–1.44289	0.14294	9.0
C_18_H_15_F2N_3_O_3_	COc1cc2ncc(C(=O)N)c(Nc3ccc(F)cc3F)c2cc1OC	0.63033	0.56239	–0.06794	–10.8
C_21_H_27_N_3_O_3_S	CCCSc1c(cnn1c2ccc(cc2)C(=O)O)C(=O)N(C)C3CCCCC3	–1.34331	–1.19841	0.14490	10.8
C_27_H_34_N_6_O_2_	CN(c1ccnc(Nc2cc(cc(c2)N3CCOCC3)N4CCCC4)n1)c5cc(CO)ccc5C	1.34117	1.48598	0.14481	10.8
C_18_H_20_Cl_2_N_2_ O_5_S	CNC(=O)OC[C@@H](C)N(c1cc(Cl)ccc1CO)S(=O)(=O)c2ccc(Cl)cc2	0.67214	0.59870	–0.07344	–10.9
C_17_H_17_N_3_O_2_	COc1cc2ncnc(Nc3cccc(C)c3)c2cc1OC	1.09029	0.97053	–0.11976	–11.0
C_20_H_20_ClNO_2_	Cc1ccc(cc1)C(=O)N2CCC(CC2)C(=O) c3ccc(Cl)cc3	1.33281	1.17617	–0.15664	–11.8

These predictions show that the GBFS-Mol2Vec model is robust across molecules of varying complexities. The predictions are generally close to the experimental values, with many differences being less than 1% in absolute terms. This indicates a relatively high accuracy of the prediction model for these compounds. The percentage differences range from −11.8% to +10.8%, showing variability in model performance across different chemical compounds. Chemical molecules with chemical formulas such as C_8_H_16_N_4_S, C_19_H_23_FN_4_O_2_, and C_9_H_13_N_3_O show very small deviations between predicted and experimental values, where molecules such as C_20_H_20_ClNO_2_ and C_18_H_20_Cl_2_N_2_O_5_S show larger deviations. No clear chemical trends are evident, although the model’s performance likely depends heavily on the chemical space represented by the training set. Moreover, the predictions do not seem to correlate with the length of the SMILES strings. Among the examples provided, the longest SMILES string, with a length of 89, has a percentage difference of +2.0% between the prediction and experimental value. Conversely, the shortest SMILES string, with a length of 11, shows a percentage difference of −5.3%. Furthermore, when comparing two distinct chemical molecules with an identical chemical formula (i.e., C_17_H_17_N_3_O_2_), it is observed that one molecule exhibits a percentage difference of 6.3%, while the other shows −11%. This variation implies that the specific chemical structures encoded by the SMILES strings provide critical information that enhances the accuracy of predictions, beyond what can be achieved by methods relying solely on chemical formulas.

In general, the results in Table 8 show that the GBFS-Mol2Vec model tends to slightly overestimate smaller values and marginally underestimate larger values of log *D*. This pattern is apparent when observing the negative values in the pred.-expt. column, where negative values suggest underestimations and positive values indicate overestimations by our model. Nevertheless, across these 60 examples, log *D* maintains a relatively balanced performance, with 27 instances showing a positive percentage difference and 33 instances showing a negative percentage difference. The MAE value on these examples is 0.05309, demonstrating that the GBFS-Mol2Vec model exhibits excellent performance, further affirming the efficacy of our proposed modeling approach.

## 4 Conclusions

This study has explored the automatic prediction of molecular properties by employing substructure vector embeddings in conjunction with a machine-learning feature selection and statistical analysis workflow, within a framework of semisupervised learning. The substructure vector embeddings were generated by adopting the methodology of Jaeger et al.^23^ This approach involves learning vector representations of molecular substructures, with the vectors oriented so that chemically similar substructures align in similar directions. The foundational principle of this method is that vectors representing closely related SMILES notations cluster together in the vector space. Moreover, our feature-selection workflow integrates a distributed gradient boosting framework along with exploratory data and statistical analyses, and strategies to mitigate multicollinearity within data sets. This methodology aims to identify and retain a subset of features that are highly pertinent to the target variable or class within a complex feature space, while minimizing feature redundancy. Subsequently, gradient-boosting trees are trained using the selected features, which are derived exclusively from the SMILES representation of a chemical molecule, and optimized using Bayesian optimization. The stages of our workflow are systematically integrated to minimize potential human bias. Consequently, feature analysis and selection, as well as model optimization and tuning processes, are conducted autonomously, eliminating the need for domain-specific knowledge at each stage of our workflow.

In previous work, we have demonstrated the application of our workflow in the realm of inorganic chemistry.^19−22^ Herein, we have pivoted our focus to applications within organic chemistry, aiming to broaden the utility of the proposed modeling methodology. We have assessed the efficacy of our modeling methodology across a diverse range of data sets, covering both regression and classification tasks within the field of organic chemistry. Our results outperform the majority of existing state-of-the-art algorithms, including those that employ graph and geometry-based models. Additionally, we extended our comparative analysis to include large language models that had been trained on a vast corpus of unlabeled data; unsupervised or self-supervised learning methods that require significant computational resources. Our GBFS-based models demonstrate competitive performance, while offering advantages in terms of balancing model accuracy with computational demands. This is particularly significant considering the urgent global imperative to realize energy sustainability; noting that alternative methodological approaches tend to require very considerable computational resources. In a case study that focuses on the prediction of lipophilicity in chemical molecules, we demonstrate the effectiveness of our modeling strategy. This case study demonstrates the usefulness of a systematic approach to feature analyses and selection over a mere reliance on complex modeling techniques.

## Data Availability

We have made the code for the feature selection, statistical analyses, multicollinearity reduction, recursive feature elimination and Bayesian optimization available at https://github.com/Songyosk/ML4SMILES. The data set used in this study are provided in *all*_*data sets*.*xlsx* as a part of the Supporting Information.

## Supporting Information Available

See the Supporting Information for the convergence, the partial dependence and the evaluation plots obtained during Bayesian optimization, in addition to the corresponding pseudocode (PDF)
The data sets utilized in this study are provided in all_data sets.xlsx as a part of the Supporting Information (XLSX)
