# Supplementary material for: Automatic Prediction of Molecular Properties Using Substructure Vector Embeddings within a Feature Selection Workflow
Source: J Chem Inf Model. 2024 Dec 23;65(1):133–52. doi: 10.1021/acs.jcim.4c01862 (PMC11733926; doi:10.1021/acs.jcim.4c01862)
Supplement: Supplementary file 1 — ci4c01862_si_001.pdf [file ci4c01862_si_001.pdf]

Supporting Information

Automatic Prediction of Molecular Properties Using Substructure Vector Embeddings within a Feature Selection Workflow

Son Gyo Jung[1,2,3], Guwon Jung[1,3,4], Jacqueline M. Cole[1,2,3,*]

[1] *Cavendish Laboratory, Department of Physics, University of Cambridge, J. J. Thomson Avenue, Cambridge, CB3 0HE, U.K.*

[2] *ISIS Neutron and Muon Source, STFC Rutherford Appleton Laboratory, Harwell Science and Innovation Campus, Didcot, Oxfordshire, OX11 0QX, U.K.*

[3] *Research Complex at Harwell, Rutherford Appleton Laboratory, Harwell Science and Innovation Campus, Didcot, Oxfordshire, OX11 0FA, U.K.*

[4] *Scientific Computing Department, STFC Rutherford Appleton Laboratory, Harwell Science and Innovation Campus, Didcot, Oxfordshire, OX11 0QX, U.K.*

*jmc61@cam.ac.uk

SI. 1 - Bayesian optimization for the GBFS model

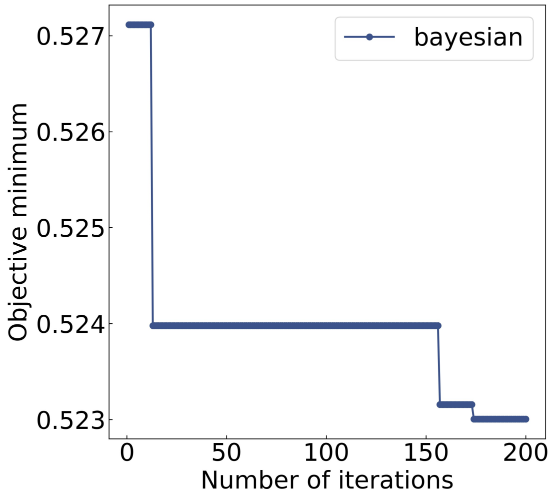

Figure S1: The convergence plot of Bayesian optimization on the validation set.

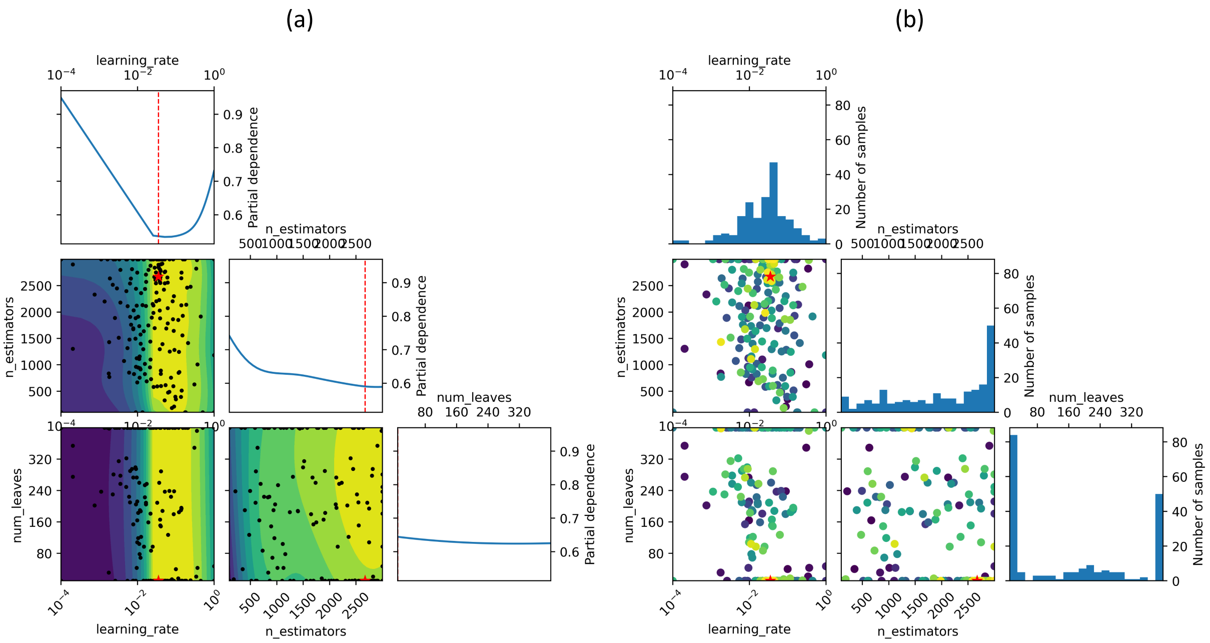

Figure S2: Bayesian optimization results of the regression model, where (a) is the partial dependence plot and (b) is the evaluation plot. The red stars indicate the values of the hyper-parameters that achieved the lowest value of the objective function. The approximate position of the objective minimum is indicated by the vertical dashed lines in red.

SI.2 Pseudo-code for Bayesian optimization

Algorithm S1: Bayesian optimization with Gaussian process prior

input: objective function f, hyper-parameter space $\boldsymbol{\theta}$, acquisition functions α,
initialization points T_{init}, maximum number of evaluation T

$y_{best} \leftarrow 0$;

for $t = 1$ **to** T_{init} **do**
    select $\boldsymbol{\theta}_t$ via randomly sampling;
    compute exact objective function $y_t \leftarrow f(\boldsymbol{\theta}_t)$;
    if $y_t > y_{best}$ **then**
        $\boldsymbol{\theta}_{best} \leftarrow \boldsymbol{\theta}_t$;
        $y_{best} \leftarrow y_t$;
    end
end

for $t = T_{init} + 1$ **to** T **do**
    build probabilistic model for f conditioned on previous observations $D_{1:t-1}$;
    compute all possible true functions using Gaussian process regression;
    optimize acquisition functions α independently based on the posterior distribution
    and propose a candidate point for each acquisition scheme
    $\boldsymbol{\theta}_{t,s} \leftarrow argmax_{\boldsymbol{\theta}}\ \alpha_s(\boldsymbol{\theta}|D_{1:t-1})$ for $s = \{PI, EI, UCB\}$;
    choose next evaluation point $\boldsymbol{\theta}_t \leftarrow argmax_{\boldsymbol{\theta}}\ softmax(\mu(\boldsymbol{\theta}_{t,s}))$;
    compute exact objective function $y_t \leftarrow f(\boldsymbol{\theta}_t)$;
    if $y_t > y_{best}$ **then**
        $\boldsymbol{\theta}_{best} \leftarrow \boldsymbol{\theta}_t$
        $y_{best} \leftarrow y_t$
    end
end

return $\boldsymbol{\theta}_{best}$

Three acquisition functions, denoted by α, each correspond to one of the following acquisition schemes: (i) Probability of Improvement (PI), (ii) Expected Improvement (EI), and (iii) Upper-Confidence-Bounds (UCB).
